# Secreted *Gaussia princeps* Luciferase as a Reporter of *Escherichia coli* Replication in a Mouse Tissue Cage Model of Infection

**DOI:** 10.1371/journal.pone.0090382

**Published:** 2014-03-04

**Authors:** Mingyu Liu, Christina Blinn, Sarah M. McLeod, John W. Wiseman, Joseph V. Newman, Stewart L. Fisher, Grant K. Walkup

**Affiliations:** 1 Biology Department, Infection Innovative Medicines, AstraZeneca R&D Boston, Waltham, Massachusetts, United States of America; 2 RAD-Transgenics, Discovery Sciences, AstraZeneca R&D Mölndal, Mölndal, Sweden; University of Iowa Carver College of Medicine, United States of America

## Abstract

Measurement of bacterial burden in animal infection models is a key component for both bacterial pathogenesis studies and therapeutic agent research. The traditional quantification means for *in vivo* bacterial burden requires frequent animal sacrifice and enumerating colony forming units (CFU) recovered from infection loci. To address these issues, researchers have developed a variety of luciferase-expressing bacterial reporter strains to enable bacterial detection in living animals. To date, all such luciferase-based bacterial reporters are in cell-associated form. Production of luciferase-secreting recombinant bacteria could provide the advantage of reporting CFU from both infection loci themselves and remote sampling (eg. body fluid and plasma). Toward this end, we have genetically manipulated a pathogenic *Escherichia coli* (*E. coli*) strain, ATCC25922, to secrete the marine copepod *Gaussia princeps* luciferase (Gluc), and assessed the use of Gluc as both an *in situ* and *ex situ* reporter for bacterial burden in mouse tissue cage infections. The *E. coli* expressing Gluc demonstrates *in vivo* imaging of bacteria in a tissue cage model of infection. Furthermore, secreted Gluc activity and bacterial CFUs recovered from tissue cage fluid (TCF) are correlated along 18 days of infection. Importantly, secreted Gluc can also be detected in plasma samples and serve as an *ex situ* indicator for the established tissue cage infection, once high bacterial burdens are achieved. We have demonstrated that Gluc from marine eukaryotes can be stably expressed and secreted by pathogenic *E. coli in vivo* to enable a facile tool for longitudinal evaluation of persistent bacterial infection.

## Introduction

The detection and quantification of bacterial colony-forming units (CFU) within animal infection models is critical for both basic research of host-pathogen interactions and pre-clinical evaluation of antibacterial agents and vaccines. However, longitudinal monitoring of CFU from *in vivo* models usually is costly and time-consuming, and requires sacrifice of animals at different time points of infection. Using luciferase-expressing genetically engineered bacteria to enable a rapid and non-destructive reporter for infection burden has been applied for a variety of bacterial infections [1]. To date, all the luminescence producing recombinant bacteria available for *in vivo* studies have used two classes of luciferase, either from luminous beetles including *Photinus pyralis* firefly luciferase (FFLuc, 62 kDa) [2], [3] and *Pyrophorus plagiophtalamus* click beetle luciferase (CBRluc, 62 kDa) [4] or luciferase from luminous bacteria including *Photobacterium* and *Vibro* genera (LuxCDABE, 77 kDa) [5]. On the other hand, luciferase from luminous marine eukaryotes such as *Gaussia princeps* has not been successfully applied for *in vivo* bacterial studies.

The *Gaussia princeps* luciferase (Gluc, 19.9 kDa) from marine copepod catalyzes the oxidation of its substrate, coelenterazine (CTZ), producing blue luminescence concomitantly. As the smallest known luciferase, Gluc in either cell-associated form or secreted form has been used for *in vivo* monitoring of a variety of mammalian cell behaviors including primary tumor growth [6], early tumor metastasis [7], cell apoptosis [8] and T cells trafficking [9]. Additionally, Gluc has also been used as reporter for *in vivo* detection of certain nonmammalian organisms such as the fungus pathogen *Candida albicans* [10] and alga *Chlamydomonas reinhardtii* [11]. Gluc demonstrates several advantages over other conventional luciferases. First, it is documented that human codon-optimized Gluc is 1000-fold more sensitive than humanized firefly luciferase (FFLuc) or humanized *Renilla reniformis* luciferase (RLuc, 34 kDa) [12]; Second, Gluc is naturally secreted in active form from *Gaussia princeps* using its native secretion signal (SS), enabling reporting from both the cells themselves and their extracelluar environment; Third, Gluc exhibits good stability under adverse conditions including low pH, hydrogen peroxide, high temperature and even β-mercaptoethanol [13], making it well suited for reporting from stress-associated *in vivo* environments that are expected within sites of infection. Last, unlike the firefly luciferase (FFluc) or bacterial luciferase (LuxCDABE) [14], [15], [16], [17], [18], [19], Gluc does not require co-factors such as ATP or FMNH_2_ for its reaction. The independence of Gluc activity from these metabolites avoids declines in detection sensitivity associated with the decreased concentration of bacterial metabolite, occuring upon stationary phase [13], [18], [19].

Despite the many potential advantages of Gluc-based bacterial reporter systems, successful attempts have only been demonstrated for *in vitro* studies, such as monitoring *Mycobacterium smegmatis (M. smegmatis) in vitro* growth under hostile conditions [13] or reporting transcription and secretion of virulence factors in the *in vitro* culture of *Salmonella enterica Serovar Typhimurium* (*S.Typhimurium*) [20]. The trials of using engineered *M. smegmatis* or *Lactococcus lactis* (*L. lactis*) to stably express Gluc are illustrative of the challenge for making *in vivo* bacterial reporter. Although Gluc was robustly expressed by *M. smegmatis in vitro*, *in vivo* imaging of *M. smegmatis* in mice lungs was not successful [2].The other endeavor of *in vivo* and *ex vivo* imaging of Gluc-producing *L. lactis* did not generate any signal above the background either [4]. In order to prepare a bacterial reporter that could benefit from the sensitivity of Gluc, the robustness of Gluc to adverse conditions, and also enable *in vivo* bacteria detection using *ex situ* sampling (eg. body fluid and plasma), we prepared an engineered pathogenic *E. coli* that can stably secrete Gluc and tested whether Gluc can function as a CFU reporter within a persistent tissue cage model of infection.

The subcutaneous tissue cage infection model [21], [22], [23] mimics deep skin bacterial infections, facilitating the study of bacterial virulence factors [24], [25], cellular immune responses [26], [27], [28], the evaluations of antibiotic efficacy, and assessment of *in vivo* emergence of bacterial resistance during antibiotic treatment of persistent infections [29], [30], [31]. For the mouse tissue cage model, a sterile, perforated teflon cylindrical tube (size: 8 mm outer diameter × 6 mm inner diameter × 20 mm length) is implanted subcutaneously (*s.c.)* on the back of mice to form tissue cages wherein interstitial fluid/tissue cage fluid (TCF) accumulates. Infection is initiated by injection of bacteria into the cage cavity *via* percutaneous puncture (*p.p.*). This model enables the study of a persistent infection, for up to 3 weeks, wherein the host cellular immune responses are in effect. As long as the initial inoculum size is controlled, tissue cage infection normally does not spread systemically but instead results in an abscess milieu within the cage [32], [33], characterized by a suppurative environment with low pH, high protein content, and stationary phase bacteria present at high density in TCF. In such a milieu, the robustness of Gluc to adverse conditions may be of particular importance. Additionally, this model provides a system to evaluate the stability of Gluc-based CFU reporter in infections of long duration. Longitudinal monitoring of bacteria in tissue cage requires multiple sampling of TCF along the infection. Doing so removes organisms from the active infection thus the sampling volume must be kept to a minimum to avoid altering bacterial growth kinetics in tissue cage. In addition, the abscess fluid (TCF) thickens with the progress of infections, making it difficult to sample a volume greater than 10 µl. Conventional CFU plating assays require at least 10 µl or more TCF for measuring bacterial burden in TCF. Therefore, developing a CFU reporter allowing the use of less TCF for bacterial detection is meaningful and important.

Here we report for the first time that the secretion of Gluc by pathogenic *E. coli* enables a convenient CFU reporter for bacterial infection in mice. Gluc empowers *in vivo* imaging of stationary phase bacteria in tissue cage infection. Furthermore, CFU burden in TCF and secreted Gluc activity in TCF correlate to a high degree. Lastly, stationary phase bacterial burden in tissue cage even can be indicated by secreted Gluc in the blood samples of mice bearing tissue cage infection.

## Results

### Secretion signal PelB derived from pectate lyase B of *Erwinia carotovora* (*E. carotovora*) efficiently promotes the secretion of Gluc by *E. coli* to culture supernatant

**Figure 1:**
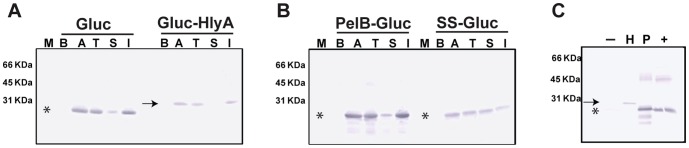
PelB secretion signal derived from pectate lyase of *Erwinia carotovora* (*E. carotovora*) promotes an efficient secretion of *Gaussia princeps* luciferase (Gluc) by *E. coli*. M: lane for protein marker, B: whole *E. coli* cell lysate before induction with IPTG, A: whole cell lysate after induction, T: total whole cell lysate after French press lysis, S: soluble fraction of lysate after French press, I: insoluble fraction of lysate after French press. Arrow: the predicted position of Gluc-HlyA on gel. Asterisk: the predicted position of Gluc, PelB-Gluc, and SS-Gluc fusion protein. For panel A and B, lysate corresponding to 10^8^ cells was loaded in each lane. (A) The expression of Gluc without secretion signal (Gluc) or Gluc with C-terminal secretion signal derived from α-hemolysin (Gluc-HlyA) was induced from pCOLDI-based vectors in *E. coli.* Expressions were detected by Western blotting using a rabbit polyclonal antibody against Gluc. (B) The expression of Gluc with N-terminal secretion signal derived from pectate lyase (PelB-Gluc) or Gluc with its native secretion signal from *Gaussia princeps* (SS-Gluc) was induced from pCOLDI-based vectors in *E. coli* and detected by the above rabbit polyclonal antibody. (C) For panel C, the bacterial culture supernatant corresponding to secreted protein from 2×10^8^ cells was loaded in each lane. −: Culture supernatant of *E. coli* expressing Gluc without secretion signal, H: supernatant of *E. coli* expressing Gluc-HlyA, P: supernatant of *E. coli* expressing pelB-Gluc, +: supernatant of *E. coli* expressing SS-Gluc. Note: The exposure time for panel A and B was shorter than for panel C. There is a gel crack in the lane of “+” which appears two bands at the position of SS-Gluc.

A total of four Gluc-derived proteins with varied secretion signaling peptides were tested in *E. coli* for their ability to promote secretion *in vitro*. These included the full length Gluc which contains the native 16 amino acid N-terminal secretion signal (SS) or Gluc with these residues removed. Two further fusion proteins were designed from the latter construct, Gluc appended to the 21 N-terminal amino acid secretion signal from pectate lyase B (PelB), and also a construct with the 60 amino acid secretion signal of Hemolysin A (HlyA) appended at the C-terminus of the Gluc protein. The cell lysates of *E. coli* harboring pCOLDI based plasmid encoding these different versions were analyzed by Western-blotting (Figure 1A and 1B). The majority of over-expressed Gluc is present in the insoluble fraction of cell lysate (comparing lane T and Lane I), yet some over-expressed Gluc is retained in the soluble fraction of cell lysate (comparing lane T and lane S). Gluc without any secretion signal is highly expressed from plasmid (comparing lane B and Lane A for Gluc in Figure 1A), but not found in the culture supernatant (Lane “-” in Figure 1C). Similar results were observed with Coomassie Blue staining (figure S1), indicating that Gluc without a secretion signal is not naturally secreted by *E. coli*. This result highlights the differing efficiency of Gluc secretion between *E. coli* and *Mycobacterium smegmatis (M. smegmatis)*, noting that almost 100% of Gluc was detected in the culture supernatant of *M. smegmatis* expressing Gluc without a secretion signal [2]. Gluc with either PelB secretion signal or native secretion signal (SS) is secreted to the culture supernatant to a greater degree than Gluc with HlyA secretion signal (comparing Lane “H” with lane “P” or lane “+” in Figure 1C). However, significant growth lag times were observed for *E. coli* cultures expressing Gluc with native secretion signal (SS) to reach the same cell density as other *E. coli* strains (Data not shown), suggesting fitness cost with this construct. Additional bands for Figure 1C lane P could reflect some degradation of the secreted PelB tagged Gluc protein. Nonetheless, the construct was selected for further study due to the greater functional activity of this clone relative to the others investigated.

### Integration of *Gaussia princeps* luciferase (*gluc*) gene into the *E. coli* chromosome

**Figure 2:**
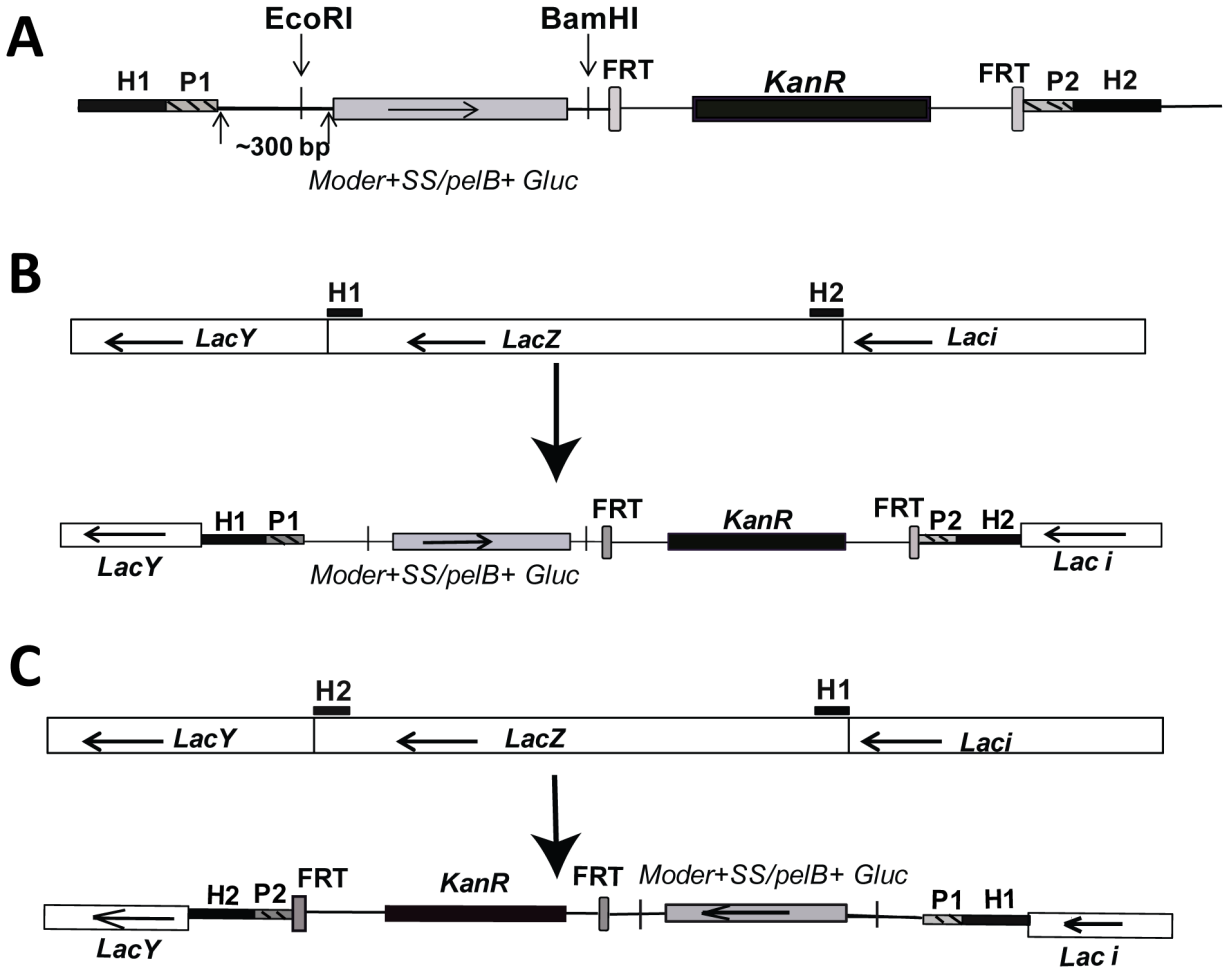
Strategy for integration of *Gaussia princeps* luciferase (*gluc*) gene into *lacZ* locus of *E. coli*. (A) Representation of the PCR fragment used for λ red integration. H1 and H2 refer to the regions homologous to the chromosome. P1 and P2 represent the priming sites for amplifying the PCR fragements from template plasmids. FRT: Flippase-mediated excision site, *KanR:* Kanamycin resistance gene, *Moder+SS/pelB+Gluc*: moderate strength promoter driven either native secretion signal (SS) tagged *gluc* gene or PelB secretion signal tagged *gluc* gene. Arrowheads show orientations of open reading frame (ORF). (B) If H1 and H2 regions correspond to the terminal region and the beginning region of *lacZ* ORF respectively, the *gluc* gene will replace the *lacZ* ORF and be integrated in the orientation opposite to the original *lacZ* ORF. *LacY: LacY* gene locus on the chromosome, *LacZ: lacZ* gene locus, *Laci: Lac I* gene locus. (C) If H1 and H2 correspond to the beginning region and the terminal region of *lacZ* ORF respectively, the integrated *gluc* will be in the same orientation as the original *lacZ* ORF.

In order to achieve a stable expression and secretion of Gluc from *E. coli*, the *gluc* gene with codon usage optimised for Gram-negative bacteria was integrated site-specifically into the *E. coli* chromosome under the control of a constitutive promoter. Three different promoters that have been reported to have varying strengths were tested [34]. Also, *gluc* with either the *pelB* or the native secretion signal (*SS*) were examined. The three promoter sequences each fused to tagged *gluc* ORFs were cloned into pSMM25, a derivative of the vector pKD4 [35], so that they were immediately 5′ to a FRT-flanked kanamycin resistance gene.Thus, a matrix of six template plasmids containing either the *pelB* tagged or the *SS* tagged *gluc* gene driven by each of the three different promoters was generated. These plasmids were used as PCR templates for generating the DNA fragments used for site-specific recombination with the chromosome. These DNA fragments were integrated into the chromosome using the λ Red recombinase [35]. In the process of cloning the *gluc* gene driven by either strong or intermediate promoters to make template plasmids, multiple mutations were repetitively observed in the promoter region of these template plasmids (data not shown), suggesting that constitutive expression of secreted forms of Gluc at elevated levels is toxic to *E. coli*. As represented in Figure 2A, the DNA fragment used for the final homologous recombination contained a moderate promoter driving either the *pelB* tagged *gluc* ORF or the *SS* tagged *gluc* ORF.

The *lacZ* locus within the genome was selected as the integration site of the *gluc* gene based on two considerations. First, LacZ is not required for *E. coli* replication and pathogenesis, avoiding the fitness cost caused by the substitution of *lacZ* with *gluc*. Second, the substitution of *lacZ* with *gluc* enables a fast X-gal Blue/White screening for targeted integration of *gluc* in *lacZ* locus. Integration of the *gluc* gene into *lacZ* locus was performed in both the opposite orientation of the *lacZ* ORF (Figure 2B) or same orientation as the *lacZ* ORF (Figure 2C). The clones obtained exhibit the *lacZ* ORF substituted with the *gluc* gene accompanied with a kanamycin drug selection cassette.

### Targeted integration of *pelB*-tagged *gluc* into the *lacZ* locus of *E. coli* yields stable secretion of Gluc

**Figure 3:**
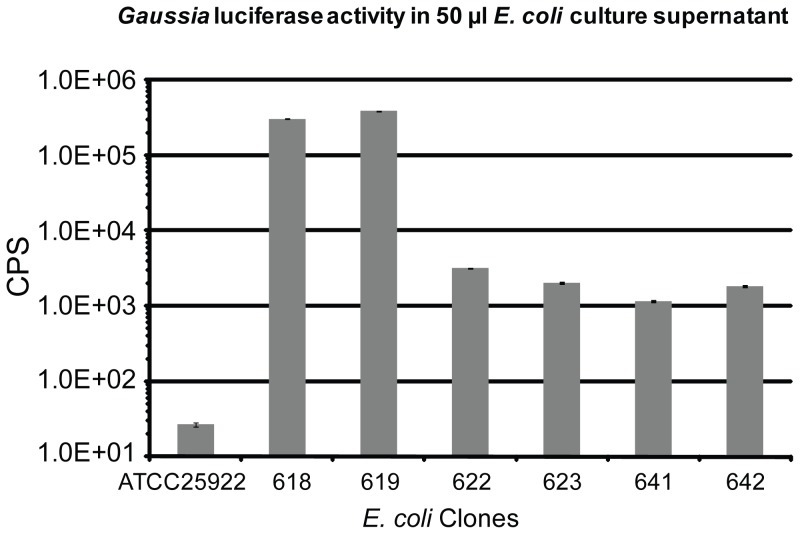
The *pelB* tagged *gluc* gene integrated in the same orientation as the original *lacZ* ORF yields a high level of secretion of Gluc to bacterial culture supernatant by *E. coli*. All *E. coli* clones were grown in LB medium without kanamycin supplement for overnight and 50 µl culture supernatant was measured for secreted Gluc activity. Note: all results have been normalized with OD_600_. CPS: photon counts per second. *E. coli* strain ATCC25922 is the negative control strain without integration of *gluc* gene on the chromosome. *E. coli* clones 618 and 619 harbor the *pelB* tagged *gluc* gene integrated in the same orientation as the original *LacZ* ORF on the chromosome. Clones 622 and 623 have the *pelB* tagged *gluc* gene integrated in the orientation opposite to *LacZ*. Clones 641 and 642 have the *SS* tagged *gluc* gene integrated in the orientation opposite to *LacZ*. Data represent mean and standard error of triplicate samples. Error bars may be too small to see for some samples.

The secretion of Gluc resulting from the *pelB* tagged *gluc* gene integrated in the same orientation as the original *lacZ* ORF is four orders of magnitude higher than the signal from the parental strain ATCC25922 and two orders of magnitude higher than the secretion from the *gluc* gene integrated in the opposite orientation (Figure 3 and Figure S2). This is presumably due to transcription from the *Lac* promoter driving expression of Gluc, in addition to the promoter fused to the *gluc* gene. Integration of the *SS* tagged *gluc* gene into *lacZ* locus was only accomplished when the orientation of *gluc* is opposite to the direction of the *lacZ* ORF (Figure 3 and S2), despite repeated attempts to integrate this fusion in the same orientation as *lacZ* transcription. This is consistent with the observation that *E. coli* expressing SS tagged Gluc from a plasmid grew extremely slowly in liquid culture, suggesting a significant fitness cost of high SS-Gluc expression and/or secretion. All the clones with the *SS* tagged *gluc* gene integrated in the orientation opposite to *lacZ* have a low level of secretion of Gluc (Figure 3 and S2).

**Figure 4:**
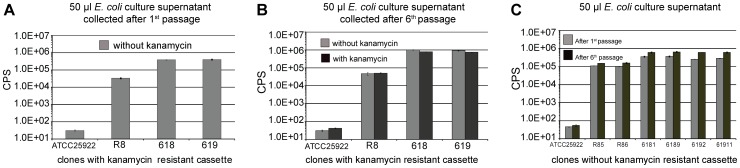
Integration of the *pelB* tagged *gluc* gene into chromosomal *lacZ* locus is stable during *in vitro* passage. All results have been normalized against OD_600_ of the culture analyzed. For all panels, data represent mean and standard error of triplicate samples. Error bars may be too small to see for some samples. CPS: photon counts per second (A) *E. coli* clones with the *pelB* tagged *gluc* gene were grown in LB medium without kanamycin for one passage and measured for the secretion of Gluc into culture supernatant. All clones in panel A harbor the kanamycin resistance gene accompanying the *pelB* tagged *gluc* gene. Clones ATCC25922, 618 and 619 are the same shown in Figure 3. Clone R8 is an *E. coli* clone with the *pelB* tagged *gluc* gene integrated in the same orientation as *lacZ*, however, the *gluc* gene is inserted into *lacZ* ORF instead of replacing *lacZ* ORF (see Experimental Procedure Table1). (B) *E. coli* clones from panel A have undergone 5 more serial passages either in media supplied with kanamycin (black bar) or without kanamycin (grey bar). Culture supernatant corresponding to the 6^th^ passage was measured for the secretion of Gluc. (C) Kanamycin resistance gene was eliminated from clone R8 to generate clone R85 and R86. Clones 6181 and 6189 are kanamycin sensitive (Kan^S^) derivatives from clone 618. Clones 6192 and 61911 are Kan^S^ derivatives from clone 619. Clones were passaged in LB medium without kanamycin. Culture supernatant collected from the 1^st^ passage (grey bar) and the 6^th^ passage (black bar) were measured for secreted Gluc activity.

We investigated the *in vitro* stability of selected integrants of Gluc tagged with PelB. First, *E. coli* clones which were shown to secrete Gluc to a high extent (Figure 3) were analyzed by *in vitro* passage experiments. Clones were grown in LB medium without kanamycin selection for one passage until an OD_600_ of about three, then diluted 1000-fold for a second passage. This protocol was followed for a total of six passages. Comparing the secreted Gluc activity in the culture supernatant of passage number one with the activity in the supernatant of sixth passage (Figure 4A,4B and S3) reveals that there is no reduction of Gluc secretion after 6 passages. Moreover, the Gluc secretion for passage number 6 is not dependent on the presence of kanamycin selection in media (Grey bar and black bar in figure 4B and S3B). Hence, the stability of the targeted integration of *gluc* gene in *lacZ* locus is independent of kanamycin selection.

The kanamycin resistance gene was then removed from three clones (R8, 618 and 619) showing stable integration of *gluc* in *lacZ* locus using the FLP recombinase to generate 6 clones as shown in Figure 4C. *In vitro* passage experiments were performed on the six clones (as described above) to confirm the stability of the *gluc* integration after elimination of the kanamycin resistance gene. These studies demonstrated that elimination of the kanamycin resistance cassette did not affect the stability of chromosomal integration of the *gluc* gene in the *lacZ* locus (passage 1 and passage 6, grey bar and black bar in figure 4C). Furthermore, the replication of the *E. coli* recombinant clone 6189 (ML6189) in either LB medium (data not shown) or in Tryptic Soy Broth (TSB) was compared against the parental strain ATCC25922 (Figure S4A). No evidence of a fitness cost was observed in these studies. Additionally, Gluc bioluminescence correlates well with bacterial density during both exponential and stationary phase of *in vitro* growth (Figure S4B).

### Optimization of the luminescence reaction catalyzed by the secreted Gluc

**Figure 5:**
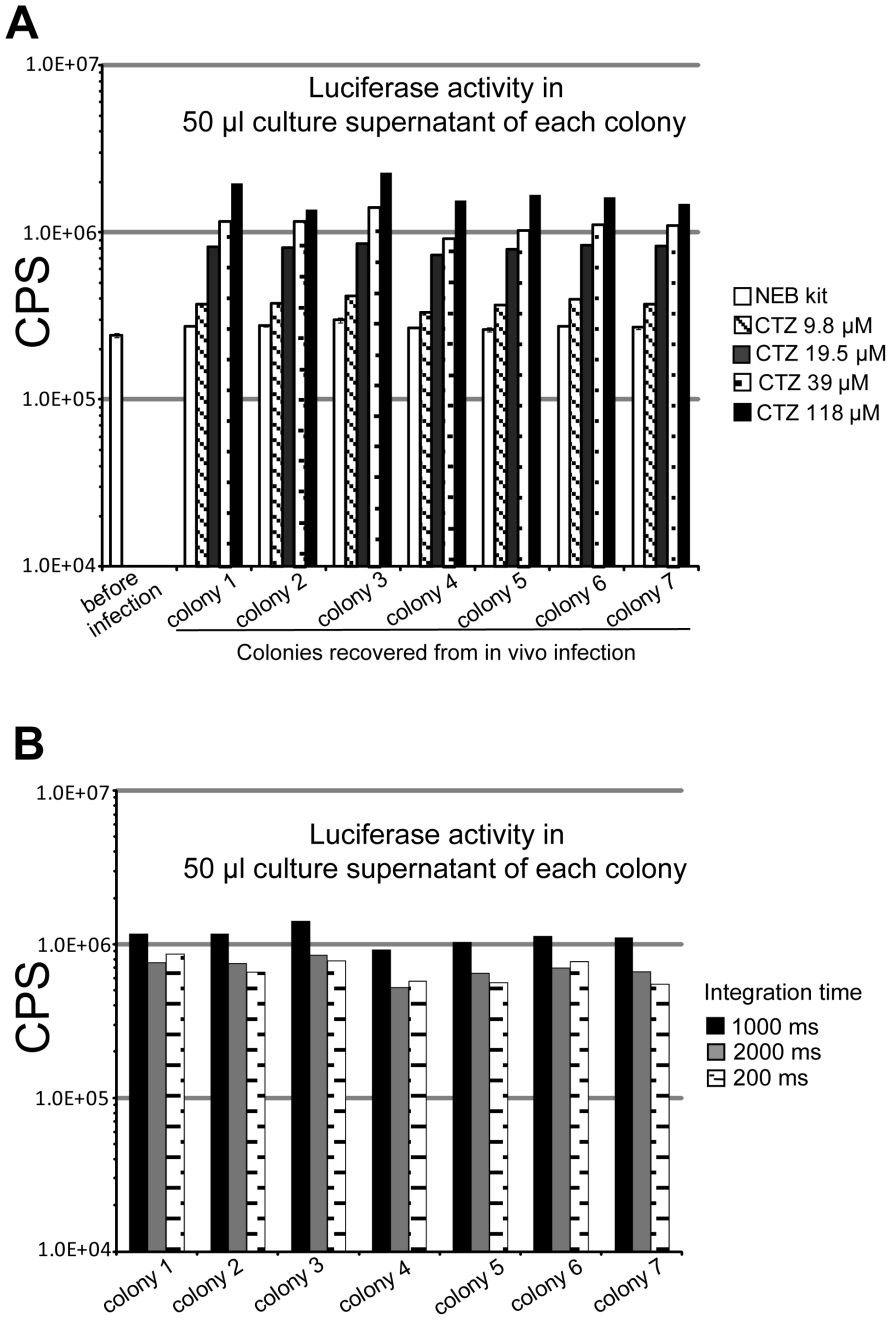
Detection sensitivity of Gluc secreted by bacteria recovered from *in vivo* infection increases with CTZ concentration, but is less affected by integration time. All results have been normalized with OD_600_. (A) *E. coli* clone 6189 (ML6189) was grown in LB medium for 16 hr and secreted Gluc in culture supernatant was measured as the “before infection” sample. *E. coli* from the above culture was used for mice thigh infection. 24 hr later, *E. coli* was recovered from infected thigh tissues and grown as colonies on Agar plate. 7 colonies were randomly picked from the plate and inoculated into LB for 16 hr. Culture supernatant of the above 7 colonies was measured for secreted Gluc activity. CTZ: coelenterazine, substrate of Gluc. For panel A, integration time for Gluc assay is 1000 millisecond (ms). (B) For panel B, 39 µM CTZ was used for Gluc assay. Overnight culture supernatant of the 7 colonies recovered from mice infection was measured for secreted Gluc activity.

In addition to the *in vitro* passage test for the stability of chromosomal integration of the *gluc* gene, we also examined the stability of the *gluc* integration after *in vivo* growth of the recombinant *E. coli* strain. Comparison of the Gluc secretion levels of the recombinant *E. coli* clone (ML6189) before and after *in vivo* thigh infection was performed using a Gluc assay kit from New England Biolab (NEB kit, white bars in Figure 5A). Colonies recovered after *in vivo* infection produced the same level of secreted Gluc activity in the culture supernatant as seen prior to *in vivo* infection (Figure 5A), verifying the stability of integration of the *gluc* gene in *lacZ* locus during the course of infection.

To maximize the luminescence signal produced by bacterial secreted Gluc, the coelenterazine (CTZ) substrate was assessed over four different concentrations up to the solubility limit (118 µM) of CTZ in biological samples relative to the NEB kit conditions (Figure 5A). Using a standard integration time of 1000 millisecond (ms), a linear dependence of luminescence produced by secreted Gluc from culture supernatant was observed with substrate concentration. Based on these results, the CTZ substrate concentration was varied as needed to obtain sufficient signal above noise in subsequent. The variation of the integration time from 200ms to 2000ms had no significant effect on luminescence production (Figure 5B). Further studies using integration time longer than 2000 ms or shorter than 200 ms did not show higher signal intensity than using 1000 ms either (data not shown). These observations in terms of integration time were independent of CTZ concentration (data not shown). Accordingly, an integration time of 1000 ms was chosen for all subsequent Gluc *in vitro* or *ex vivo* assays.

### *In vivo* imaging of Gluc generated luminescence

To assess whether the Gluc secreting capability of *E. coli* recombinant clone could endure a typical tissue cage infection course (greater than a week), mice with tissue cages infected by strain ML6189 were imaged at both 11 and 18 days post-infection, when CFU burdens are fully established at ≥10^8^ CFU/ml tissue cage fluid (TCF). The background level of luminescence signal was determined by imaging mice infected with ATCC25922 parental strain.

**Figure 6:**
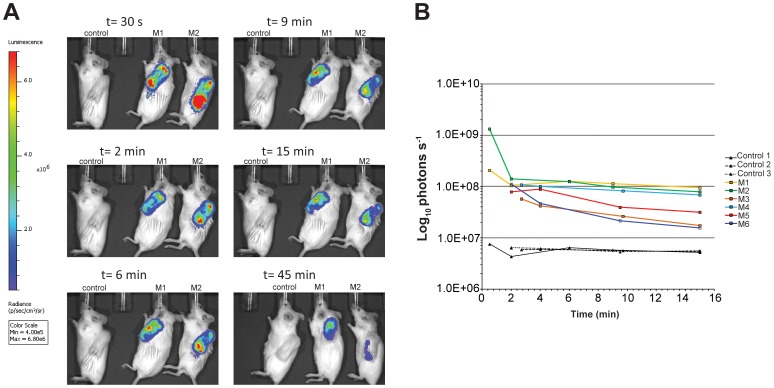
Live animal imaging can detect Gluc activity in mouse tissue cages infected with the recombinant *E. coli*. Mouse tissue cages were inoculated with either 10^3^ CFU of *E. coli* strain ML6189 (from M1 to M4) or 10^5^ CFU of ML6189 (M5 and M6) for 11 days before imaging. Control mice were inoculated with either 10^3^ CFU of *E. coli* strain ATCC25922 (control 1 and control 2) or 10^5^ CFU of ATCC25922 (control 3) for 11 days. (A) Two representative mice (M1 and M2) are shown. Photos were taken and presented as pseudo-color images indexed to luminescence intensity at a given location. Unit for color scale bar is photons second^−1^ cm^−2^ steradian^−1^. Images were acquired at time points of 0.5, 2, 6, 9, 15 and 45 min after CTZ substrate injection using an integration time of 16 seconds for all acquisitions. Note: After imaging at 15 min post-substrate injection, mice were put back to cages prior to being imaged again at 45 min post-substrate injection. (B) Total luminescence (unit as photons second^−1^) at each imaging time point was quantified by the region of interest tool in the Living Image software program (version 4.3.1).

Three routes of substrate administration including intraperitoneal (*i.p.*), tail vein/intravenous (*i.v.*) and intra-tissue cage (*i.t.)* were tested using a high concentration of CTZ (4mg/kg body weight), while avoiding solubility issues of CTZ in physiological fluid. Neither *i.p.* nor *i.v.* injection of substrate produced sufficient luminescence signal for imaging detection in tissue cages (Data not shown). In contrast, *i.t.* injection gave rise to prominent luminescence signal immediately after substrate administration (Figure 6A, t = 30s). The intensity differences that resulted from different routes of substrate administration most likely reflect the impediment to perfusion of CTZ from the periphery to the tissue cage environment.

The time dependence of the luminescence signal was assessed. The most vivid image was observed immediately following substrate administration (t = 30 sec post CTZ injection), followed by a reduced level plateau stage (2–6 min post CTZ injection) that gradually decreases over time (6–30 min post CTZ injection) where the signal decays by ∼30–70% with signal in some mice returning to background level (Figure 6 and data not shown). The rapid decay of luminescence hampers the quantitation of imaging results acquired immediately after CTZ administration due to the time requirement for positioning mice under the camera. Imaging at the plateau of the luminescence reaction (2–5 min post CTZ administration) can decrease the variability of signals among different mice but results in a 2–10 fold reduction in peak signal (Figure 6B and data not shown), thus reducing the sensitivity of luminescence imaging. In addition, imaging at plateau stage generated a signal to background ratio of one log (Figure 6B), a window which maybe too small to differentiate tissue cages with log scale difference in terms of CFU burden. Based upon the above considerations, we next measured the secreted Gluc activity *ex vivo* by taking samples of fluid from the tissue cage.

### Secreted Gluc activity in tissue cage fluid (TCF) and CFU burden of the recombinant *E. coli* are correlated

**Figure 7:**
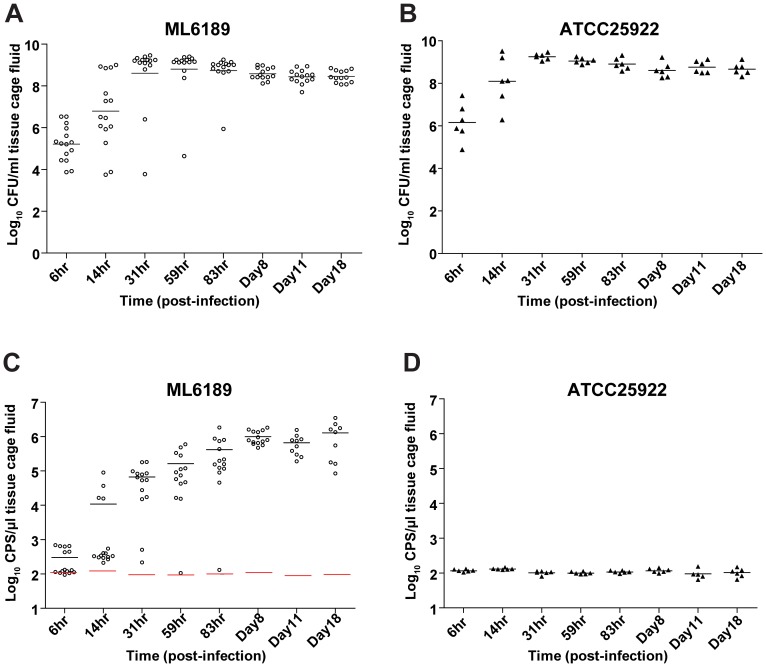
CFU burden of the recombinant *E. coli* in tissue cage fluid (TCF) and secreted Gluc activity in TCF correlate to a high degree for the established tissue cage infection. Mouse tissue cages were inoculated with 10^5^ CFU of the recombinant *E .coli* strain ML6189 expressing Gluc or the parental *E. coli* strain ATCC25922. TCF was collected at different time points and analyzed by both CFU plating and measurement of secreted Gluc activity (details seen Experimental Procedures). CPS: photon counts per second. For all panels, the mean value for each group of samples is represented by a black bar. Data from two independent experiments were pooled. (A) CFU burden of ML6189 in TCF at different time points during 18 days of tissue cage infection. (B) CFU burden of ATCC25922 in TCF at different time points along 18 days of tissue cage infection. (C) Secreted Gluc activity in TCF at different time points of ML6189 infection. The red bars represent mean value of the background Gluc signal calculated from data in panel D. Note: for ML6189 infection, 4 mice (day 11) and 5 mice (day 18) were removed from the group for imaging experiments and were excluded from quantification of Gluc activity in TCF (panel C). (D) Secreted Gluc activity in TCF at different time points of ATCC25922 infection.

To test whether Gluc secretion induced a fitness cost *in vivo*, the growth rate and time dependency of CFU burden were assessed for the Gluc secreting strain ML6189 and the parental strain ATCC25922 over 18 days in the tissue cage model. In addition, both CFU in TCF and the secreted Gluc activity present in TCF were monitored simultaneously over 18 days of tissue cage infection to profile the correlation between CFU burden and detection of secreted Gluc. For tissue cages inoculated with 10^5^ CFU, both strains demonstrated the same CFU increase (about 3 logs) within the first 3 days and the maximal CFU burden was reached by day 3 (59 hr) post-infection (≥10^8^ CFU/ml TCF). At this time point, there was no statistically significant difference between the two strains in establishing the infection (ML6189: 13/14 mice, 93%; ATCC25922: 6/6 mice, 100%; Figure 7A and 7B). More importantly, the established CFU burden of ML6189 remained at a constant density until day 18, showing that the infection persistence was not impacted by the expression and secretion of Gluc. Similar results were obtained in experiments with a lower initial inoculum (10^3^ CFU per tissue cage), where the infections in most mice were fully established with both strains by day 3 (Figure S5A and S5B). Further, both strains had a persistent *E. coli* burden for the remaining course (18 days) of the tissue cage infection (Figure S5A and S5B). In all cases, the infections remained localized to the tissue cage and there was no evidence of systemic dissemination (data not shown). Thus, *in vivo* replication of the Gluc secreting strain is not compromised relative to the parental strain.

For *ex vivo* measurement of the secreted Gluc in TCF, the equivalent of as little as 1 µl of TCF can be used for quantitation of Gluc activity. In these studies we regularly sampled 20 µL of TCF to enable simultaneous CFU plating, however luminescence measurements involved sample dilution equating to 1 µL of TCF sample per test well. The background level of Gluc activity in TCF was determined by measuring TCF from mice infected with ATCC25922 parental strain (Figure 7D). For tissue cage infections initiated with 10^5^ CFU, the increase of Gluc activity in TCF correlates with the growth of CFU (Figure 7A and 7C). First, there is 3 logs increase of Gluc activity within the first 3 days (59 hr), the same magnitude of increase as the CFU density. Second, Gluc activity levels off at day 4 while the growth, as measured by CFU, levels off at day 3. Importantly, Gluc activity persists from the 83 hr time point throughout the course of the infection (18 days). It should be pointed out that the mice that did not show Gluc activity above background across all time points are the same mice which failed to establish a robust infection of ≥10^8^ CFU/mL TCF. For tissue cage infections initiated with 10^3^ CFU, a correlation between secreted Gluc activity in TCF and CFU burden in TCF was also observed (Figure S5A and C) except that the Gluc activity leveled off later than 83 hours post inoculation. Careful investigation of paired CFU and luminescence measurements from individual samples taken during the first three time points (≤31 h, Figure 7 and S5) showed that Gluc activity in TCF were above the background level when CFU reaches the density of about 10^6^ CFU/mL TCF. The tissue cage used in these studies holds a maximum of 300 µl TCF *in vivo,* suggesting that the minimal number of bacteria in TCF enabling CFU detection *via* secreted Gluc is less than or equal to 3×10^5^ CFU per tissue cage. Furthermore, the *ex vivo* luminescence signal rises significantly above background after the infection burden is fully established (≥10^8^ CFU/ml TCF or 3×10^7^ CFU per tissue cage).

*Ex vivo* measurement of secreted Gluc for established tissue cage infection presented a signal to background ratio of three logs, a quantification window larger than the one generated by imaging. More importantly, measuring samples *in vitro* with a luminometer equipped with a substrate injector avoids the timing complications encountered in whole-animal imaging, thereby aiding sensitivity of detection. Lastly, *ex vivo* measurements required much less CTZ substrate than the amount infused into whole animal for imaging experiment. In summary, *ex vivo* measurements of Gluc in TCF requires sampling of TCF, but provides a sensitive method of detecting secreted Gluc as a reporter for *E. coli* replication.

### Secreted Gluc can be detected in plasma samples and serve as an *ex situ* indicator for the localized tissue cage infection

**Figure 8:**
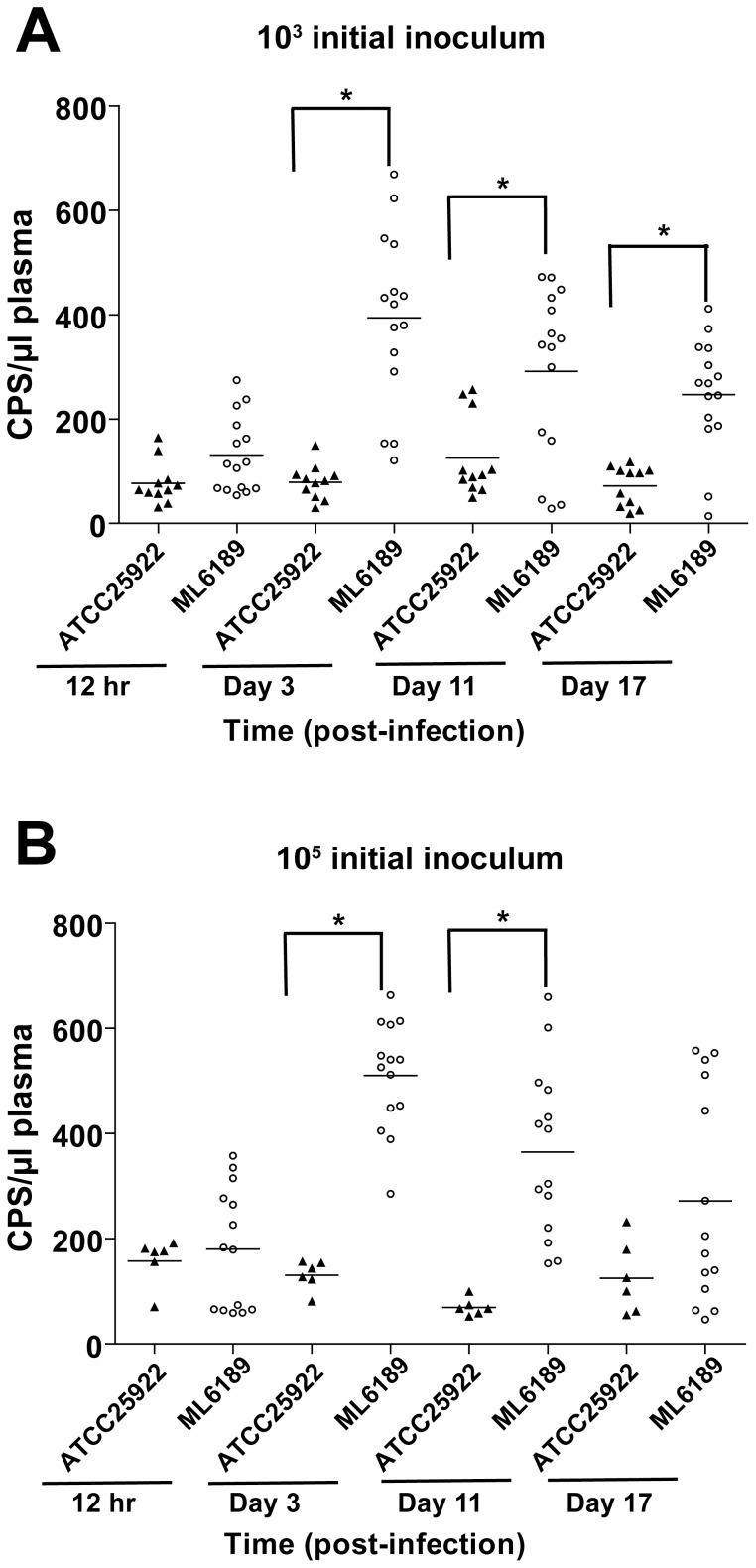
Secreted Gluc permeates from the established tissue cage infection loci to the blood stream and serves as an *ex situ* indicator for the localized tissue cage infection. Mouse plasma was collected at the denoted time points during tissue cage infection and measured for Gluc activity present in plasma (details seen Experimental Procedures). (A) Mouse tissue cages were inoculated with 10^3^ CFU of ML6189 or ATCC25922 at the beginning of infection. (B) Mouse tissue cages were inoculated with 10^5^ CFU of ML6189 or ATCC25922 at the beginning of infection. Unpaired and two tailed Student's *t*-test was used for all two comparisons with one variable (indicated by the connected lines in the figure), *P*<0.01 was considered as statistically significant and marked by “*”. For all panels, the mean value for each group of samples is represented by a black bar. Data from two independent experiments were pooled.

We sought to test whether the Gluc secreted by *E. coli* could be detected in the circulating blood and used for quantification of CFU burden in tissue cage infection loci. Tissue cage infections initiated with either 10^3^ CFU (Figure 8A) or 10^5^ CFU (Figure 8B) were measured for plasmatic Gluc activity at four time points during a tissue cage infection (details seen Experimental Procedures). At an early time point of infection (12hr), there was no significant difference between ML6189 (Gluc secreting strain) infection and ATCC25922 (parental strain) infection in terms of detectable Gluc activity in plasma. However, once the tissue cage infections proceeded into stationary phase at day 3 (59hr), the mean value of plasmatic Gluc activity was about 4-fold higher for the Gluc secreting strain (Figure 8A, B). The detection of Gluc activity in plasma samples of tissue cage infection proved that bacterial secreted Gluc can serve as a blood indicator for a well-established localized infection. For the persistent stage of infection from day 3 to day 18, the plasmatic Gluc activity correspondingly remained above the background.

As expected, the Gluc signals in plasma samples are lower than the ones in TCF for the same time points (Figure 7, 8 and S5). Although this limits the use of blood Gluc activity as a quantitative reporter for bacterial replication in tissue cage, it is useful as a qualitative blood indicator for advanced stages of tissue cage infection. Overall our results demonstrated that there are three ways of using Gluc secreted by *E. coli* to monitor bacterial replication within a tissue cage, including *in vivo* imaging, as well as *ex vivo* measuring of Gluc activity in either tissue cage fluid or plasma samples.

## Discussion

Despite the extensive use of Gluc expression in mammalian cells for monitoring *in vivo* cell behavior [6], [7], [8], [9], [36], successful *in vivo* applications of this reporter for prokaryotic organisms have not been reported to date. Other researchers have explored *M. smegmatis* and *L. lactis* systems expressing Gluc, but did not observe luminescence signals above the background for *in vivo* imaging of bacteria [2], [4]. Additionally, for these systems, no Gluc signal was detected using *ex vivo* assays such as measurement of secreted Gluc in blood samples of the infected animal [2], [4]. Herein we report that GLuc expressed and secreted by pathogenic *E. coli* can be used as a reporter for monitoring localized *E. coli* tissue cage infection *via* either *in vivo* luminescence imaging on whole animals or *ex vivo* measurement of mouse interstitial fluid samples and blood samples. Compared to the traditional method of CFU quantitation, which requires outgrowth in media over long periods (hours-days), the potential for real time quantitation of *in vivo* bacterial burden using this luciferase reporter is especially useful.

To produce an engineered *E. coli* that stably secretes Gluc, it was necessary to optimize both the expression promoter and the secretion tag. Our trials of making plasmids expressing Gluc driven by strong promoters repeatedly induced multiple mutations in the promoter region. In addition, high expression and or secretion of Gluc with its native (eukaryotic) secretion signal by pCOLDI expression plasmid severely affected the growth of *E. coil* in culture. These results are in contrast with what seen in tumor cells where Gluc with its native secretion signal driven by the strong CMV promoter demonstrated a consistent, high expression and secretion of Gluc [12], [37]. The discrepancy between tumor cells and bacteria suggested that bacteria cannot tolerate the high levels of constitutive Gluc expression, particularly when Gluc has its native secretion signal. To find the balance between bacterial fitness and sufficient Gluc expression for *in vivo* detection, a promoter reported to have moderate strength [34] was selected for driving expression of the *E. coli* codon-optimized *gluc* gene. In addition, it was found that plasmid encoding Gluc in *Lactobacillus plantarum* (*L. plantarum*) was not stable [4], albeit the decent expression of Gluc from the episomal plasmid in *L. plantarum*. Hence, in contrast to expression of Gluc from plasmid in either *M. smegmatis* or *L. lactis* [2], [4], the *gluc* gene was stably integrated on the *E. coli* chromosome in the non-essential *lacZ* locus (Figure 2, 3 and 4). Recent reports indicate that the Gluc native secretion signal negatively affects Gluc protein production and/or activity in *E. coli* [38], [39]. Therefore, the conventional bacterial PelB secretion signal was used for promoting Gluc secretion by *E. coli* (Figure 1). Traditionally, the N-terminal 22 amino acid leader sequence of PelB directs the protein to the periplasmic space, after which this signal sequence is cleaved. We found that the PelB secretion signal could promote the delivery of Gluc across the *E. coli* outer membrane to the culture supernatant while retaining high activity of Gluc (Figure 1C, Figure 3 and 4). The above combination of correct promoter strength, bacterial secretion signal and stable chromosomal integration enabled the isolation of an engineered virulent *E. coli* strain that stably expresses Gluc within animal infection models.

Our results showed that Gluc expressed by *E. coli* could serve as a reporter for monitoring tissue cage infection by *in vivo* luminescence imaging (Figure 6). While this result represents a significant advance, the practical limitations of the imaging experimental procedures, including frequent substrate injection and anesthesia, present challenges in monitoring a large cohort of animals at short time intervals. In addition, it is well-known that the luminescence propagation through living tissue can cause compromised sensitivity, reduced spatial resolution and accuracy [40]. The *ex vivo* assessment of secreted Gluc in TCF and blood samples as reporter for bacterial burden addresses the above limitations and therefore has significant advantages (Figure 7 and 8). Importantly, the window of signal to background ratio provided by *ex vivo* measurement of Gluc activity in TCF (3.5–4 logs) is not only bigger than the range (1–1.5 logs) provided by Gluc *in vivo* imaging (Figure 6, 7 and S5), but also wider than the window showed by *in vivo* imaging of FFluc expressing *M. smegmatis* (2–2.5 logs) [2].

Secreted Gluc generates bright *in vivo* and *ex vivo* luminescence during the plateau stage of tissue cage infection (Figure 6, 7 and S5), in other words, there was no evidence of decreased Gluc luminescence for *in vitro* (Figure S4B) and *in vivo* cultures (Figure 7, S5) that achieved stationary phase, supporting the idea that Gluc is well suited for monitoring infection models focusing on stationary phase bacteria due to the independence of Gluc from bacterial exponential phase metabolites (ATP or FMNH2). As expected, the Gluc reporter enabled CFU quantification with the equivalent of as low as 1 µL of TCF (Figure 7 and S5), demonstrating the required sample volume for Gluc measurement is small, which should minimize the impact on bacterial growth kinetics upon repetitive TCF sampling. Additionally, the difficulty of sampling viscous TCF can be overcome by minimized sampling volumes.

The minimal density of *E. coli* capable of being detected by *ex vivo* assessment of Gluc in TCF is around 10^6^ CFU/ml TCF, corresponding to an absolute number of ≤3×10^5^ CFU per tissue cage (Figure 7 and S5). This detection sensitivity compares favorably to other non-Gluc based luciferase-derived bacterial reporters. For example, it has been shown that the minimal density of Click Beetle luciferase (CBRluc) expressing *L. plantarum* required for *ex vivo* detection of CBRluc in feces is 10^5^ CFU/100 mg feces [4]. Additionally, the minimal number of FFluc expressing *M. smegmatis* enabling *in vivo* imaging is around 6.6×10^6^∼1.4×10^7^ CFU per lung [2].

Our results advance the understanding of using Gluc as a reporter for *in vivo* bacterial infection. However, some concerns should be resolved to broaden the applications. Reporting CFU *via* secreted Gluc in TCF requires a burden of ≥10^6^ CFU/mL TCF to enable detection above the background luminescence. Therefore, the CFU burden at 14 hr post infection (hpi) was not well represented by Gluc activity in TCF for all animals sampled (Figure 7 and S5). Individual animals at this time point with Gluc signals above the background corresponded to those animals with ≥10^6^ CFU/mL TCF. This effect is more prominent for the infections initiated with 10^3^ CFU than the ones with 10^5^ CFU, consistent with the requirement of the lower innocula to grow further to achieve 10^6^ CFU/mL. Further optimization of the detection of Gluc in biological samples may be able to address this issue. However, Gluc activity in TCF indeed showed the similar magnitude of increase with CFU burden in TCF for the later time points (59 hr and later, Figure 7 and S5), indicating that the current detection limit does not pose a problem for the established phase of infection, which has frequently been the main focus of tissue cage infection model.

All previous successful trials using secreted Gluc present in blood as reporter for *in vivo* processes are from oncology studies. Published work on Gluc-secreting human glioma cells and breast adenocarcinoma cells have shown a dynamic range with 2–3 orders of magnitude for quantification of *in vivo* implanted tumor cells by blood Gluc activity [6], [7]. However, such a broad dynamic range was not observed for detecting blood Gluc activity produced from bacteria in our tissue cage studies. This is not particularly surprising since the bacterial infection remained localized to the tissue cage, thereby presenting another permeation barrier for secreted Gluc diffusing from the Gluc production site to the blood stream. An example highlighting the impact of the location of Gluc production lesion on dynamic range is that Gluc secreted by human glioma cells implanted subcutaneously in mice provides a dynamic range of 4 orders of magnitude in blood, while the same cells implanted in mice brain only generates a range of 1 order of magnitude in blood [6]. Further, Gluc secretion by human breast adenocarcinoma cells implanted in mice mammary fat pad produces a dynamic range of about 1.5 orders of magnitude in blood [7]. These results suggest that the signal intensity of Gluc in blood is affected by a variety of disease parameters including the duration and location of the Gluc-producing lesion as well as the level of Gluc expression by the pathogen.

Expression and secretion of Gluc with its native secretion signal at a high level *via* the strong CMV promoter were tolerated by tumor cells, which may be due to the fact that tumor cells naturally lack many replication checkpoints [6], [12]. In contrast, we and others have showed that expressions of Gluc at high levels or with its native secretion signal severely compromise bacterial fitness [4], [39]. These results suggest that it will not be straightforward to boost the signal intensity of Gluc in blood *via* increasing expression of Gluc from bacteria. An alternative approach such as optimizing the detection method of Gluc in biological samples, especially in blood/plasma samples, is indicated. It was observed that the luminescence signal produced by Gluc increases with increasing substrate concentration for bacterial culture supernatant samples (Figure 5) [2], and this trend was the same for measurements of Gluc in TCF and blood samples (Data not shown). This suggests that using high concentration of CTZ substrate reduces the interference of Gluc activity by other molecules in biological samples. However, solubility limitations of CTZ in biological samples and physiologically relevant buffers restrict the maximal CTZ concentration (e.g. 118 µM in these studies). Therefore, improvements in the physical properties of CTZ may enhance the detection of Gluc in biological samples. To this end, a new chemical variant of CTZ with improved aqueous solubility was recently reported to show enhanced light output for *in vivo* imaging and *ex vivo* blood assay, albeit it has a faster luminescence decay rate [41]. Additionally, there have been successes in engineering Gluc for improved kinetics and spectral-enhancements that may provide further sensitivity [39], [42], [43], [44]. Taken together, these advances demonstrate the potential for further optimization of the bacterial secreted Gluc as a fast and sensitive reporter for longitudinal evaluation of bacterial infection.

## Experimental Procedures

### Ethics Statement

This research was performed by strict following the guidelines of Public Health Service Policy on Humane Care of Laboratory Animals and the mouse research protocol used here was approved by the Institutional Animal Care and Use Committee of AstraZeneca (IACUC Permit Number: 11-01-i). Mice were anesthetized with intraperitoneal (*i.p.*) injection of 80 mg/kg Ketamine and 10 mg/kg Xylazine during tissue cage surgery, and all efforts were made to minimize suffering.

### Bacterial strains and Culture

All strains/clones used and or generated in this study can be found in Table 1 and List S3 of File S1. For assay of Gluc luciferase secretion *in vitro*, *E. coli* clones were grown in either LB medium or Tryptic soy broth (TSB) medium for 16 hr at 200 rpm at 37°C. The culture was centrifuged for 2 min at 16,000×*g* and the supernatant collected. For cultivation of bacteria for mouse infections, *E. coli* clone ML6189 or ATCC25922 was grown in TSB medium for 16 hr at 200 rpm at 37°C. Then the overnight culture was diluted 1∶10 in TSB, the OD_600_ determined. The calculated volume of the overnight culture was pipetted into the appropriate volume of saline to obtain the target inoculum of either 10^3^ CFU/100 µl per tissue cage, 10^5^ CFU/100 µl per tissue cage or 10^6^ CFU/100 µl per mouse thigh.

**Table 1:** *E. coli* Strains/Clones used in this study.

Strain	Relevant characteristic(s)	Source/Reference
ATCC25922	Kanamycin-sensitive (*Kan^S^)*	[45]
618	*pelB* tagged *gluc* gene replacing chromosomal *lacZ* ORF with the same orientation as original *LacZ*, *Kan^R^*	This study
619	same as the above	This study
622	*pelB* tagged *gluc* gene replacing chromosomal *lacZ* ORF with the orientation opposite to original *LacZ, Kan^R^*	This study
623	same as the above	This study
641	native secretion signal (*SS*) tagged *gluc* gene replacing chromosomal *lacZ* ORF with the orientation opposite to original *LacZ, Kan^R^*	This study
642	same as the above	This study
R8	*pelB* tagged *gluc* gene inserting into chromosomal *lacZ* ORF with the same orientation as *lacZ, Kan^R^*	This study
6181	*pelB* tagged *gluc* gene replacing chromosomal *lacZ* ORF with the same orientation as original *LacZ*, *Kan^S^*	This study
6189	same as the above	This study
6192	same as the above	This study
61911	same as the above	This study
R85	*pelB* tagged *gluc* gene inserting into chromosomal *lacZ* ORF with the same orientation as *lacZ, Kan^S^*	This study
R86	same as the above	This study

### Western Blotting

*E. coli* harboring pCOLDI construct was grown in LB medium at 37°C with shaking at 200 rpm until OD_600_ reached 0.5. A portion of bacteria was pelleted (5,000×*g*, 10 min) to provide the pre-induction sample (“B” in figure 1). The rest of the culture was cooled down on ice prior to being transferred to 18°C and induced with 1 mM IPTG overnight with shaking at 200 rpm. A second portion of culture was pelleted for the post-induction sample (“A” in figure 1) and the supernatant saved as the post-induction supernatant. The remaining culture was pelleted, resuspended in lysis buffer (20 mM Tris pH 7.5, 1 mM EDTA, 5% glycerol,) and lysed by French press. A portion of lysate was saved as total lysate (“T” in figure 1). The remaining sample was subjected to 15,400×*g* centrifugation for 30 min and the supernatant saved (sample “S” in figure 1). The pellet was retained as the insoluble fraction (“I” in figure 1). Aliquots corresponding to 1×10^8^ cells were loaded into each well of protein gel and Gluc was detected by Western blotting using a rabbit polyclonal antibody against Gluc (NEB, Ipswich, MA). The post-induction supernatant samples were concentrated by chloroform-methanol precipitation [46]. Aliquots corresponding to the supernatant from 2×10^8^ cells were loaded into each well of protein gel and detected by Western as described above.

### Cloning and generation of chromosomal integration

All primers used in this study can be found in List S1 of File S1 and the plasmids used are presented in List S2 of File S1. *E. coli* codon-optimized *gluc* open reading frame (ORF) without secretion signal (Gluc), with HlyA secretion signal (Gluc-HlyA), with PelB secretion signal (PelB-Gluc) and with SS secretion signal (SS-Gluc) were synthesized (Epoch Life Science, Missouri, TX) and cloned in pBlueScript (II)SK(-) [47] to generate GS4624-1,2,4 and GS50101 respectively (List S2 in File S1). Promoter sequences which have previously been reported to have strong, intermediate and moderate strength [34] were also synthesized and cloned in pBlueScript (II)SK(-) respectively to generate GS50065-1,2,3 (List S2 in File S1). Sequences for Gluc, Gluc-HlyA, PelB-Gluc and SS-Gluc were inserted into pCOLDI [38] through NdeI and BamHI restriction digestion and ligation to generate pCOLDI (Gluc), pCOLDI (Gluc-HlyA), pCOLDI (PelB-Gluc), pCOLDI (SS-Gluc) (List S2 in File S1). Moderate promoter driven either the SS tagged *gluc* or the PelB tagged *gluc* was cloned into pSMM25 through a blunt-sticky ligation using Eco53kI/BamHI digested promoter driven *gluc* construct and BmgI/BamHI digested pSMM25 to generate two template plasmids, pJWW3 and pJWW6 respectively (List S2 in File S1).

pSMM25 is the derivative of pKD4 [35], for making plasmid pSMM25 from pKD4, an Eco RI site and a Bam HI site were introduced just upstream of the 5′ FRT site flanking the kanamycin resistance gene. These restriction sites were created in pKD4 by performing two rounds of mutagenesis using the QuickChange Mutagenesis kit from Stratagene (La Jolla, CA) according to the manufacturer's protocol. Briefly, first the BamHI site was created using mutagenic primers Smm105 and Smm106. The Eco RI site was subsequently introduced using primers Smm107 and Smm108. Following each round of mutagenesis, the DNA was transformed into chemically competent PirPlus DH10bpir116 cells (Open BioSystems, Huntsville, AL) to support replication of the *pir*-dependent R6kg replication origin of the pKD4 parent plasmid. The presence of the introduced restriction sites were verified by restriction digest of purified plasmid with Eco RI and Bam HI.

Primers, either the pair of FlacZ_ForwardL_MYL and FlacZ_ReverseL_MYL or the pair of OlacZ_ForwardL_MYL and OlacZ_ ReverseL_MYL, were used to amplify *gluc* (with *ss* tag or *pelB* tag) together with a kanamycin resistance cassette from pJWW3 or pJWW6. Integration of either the *pelB* tagged *gluc* or the *ss* tagged *gluc* into *lacZ* locus of ATCC25922 chromosome by λ red recombinase strategy was performed as described previously [35]. The DNA fragment produced from the primer pair of FlacZ_ForwardL_MYL and FlacZ_ ReverseL_MYL was used to integrate *gluc* in the same orientation as the original *lacZ* ORF. The DNA fragment produced from the primer pair of OlacZ_ForwardL_MYL and OlacZ_ReverseL_MYL was used to integrate *gluc* in the orientation opposite to the original *lacZ* ORF. Successful targeted integration events were selected for white colonies on X-gal plates, kanamycin resistance (Kan^R^), and ampicillin sensitivity (Amp^S^ for the expulsion of the λ red recombinase expressing helper plasmid) and were validated by PCR tests using locus specific primers (List S1 in File S1). The above Kan^R^ integrants were transformed with pCP20 for elimination of the kanamycin resistance selection marker on chromosome as described previously [35].

### Mice and Tissue cage model

All animal protocols were conducted under approved institutional guidelines (IACUC protocol number 11-01-i). Mice were kept in a specific pathogen free (SPF) facility of AstraZeneca Boston R&D. Eight weeks old female CD1 mice (Charles River Lab, Boston, MA) were anesthetized with intraperitoneal (*i.p.*) injection of 80 mg/kg Ketamine and 10 mg/kg Xylazine. When Stage III anesthesia was achieved, as determined by lack of pedal reflex, buprenorphine (0.1 mg/kg) was subcutaneously (*s.c*.) administrated to mice. A sterile tissue cage was *s.c.* implanted on the mouse back using aseptic techniques [27], [48]. The tissue cages were closed polytetrafluoroethylene Teflon cylinder with frequently spaced 0.2-mm holes on the wall. Tissue cages implanted had dimensions of 20 mm length by 8 mm external diameter (6 mm internal diameter) accomodating an internal volume of 600 µl. Two weeks after surgery, sterility of implanted cage was verified by the absence of colony on Tryptic Soy Agar (TSA) plates when plating about 15 µl of tissue cage fluid (TCF) on the plate.

### Sampling of tissue cage fluid (TCF) and plasma from infected mice

Mice were anesthetized by inhalation of isofluorane and about 20–40 µl interstitial fluid (TCF) in tissue cage was drawn by percutaneous puncture (*p.p.*) using a sterile 25-gauge needle. For determination of CFU burden in TCF, 20 µl of TCF was 1∶10 serially diluted in saline and plated on TSA plates. Colonies were counted after 16 hr incubation at 37°C. The remaining TCF sample was subjected to a 4°C centrifugation of 7,500×*g* for 15 min to yield a clear supernatant that was frozen at −80°C until use. About 50–100 µl blood was drawn from mice by submandibular bleeding [49] and transferred into K_2_EDTA containing plastic tubes (Becton Dickinson & Company, Franklin Lakes, NJ) to avoid clotting. Blood then was centrifuged at 2,000×*g* for 25 min at 4°C to harvest plasma sample that was frozen at −80°C until use.

### *Gaussia* luminescence *in vivo* imaging

*Gaussia* luciferase substrate, coelenterazine (CTZ-10, Gold BioTechnology, St. Louis, MO) was reconstituted in methanol to a concentration of 11.8 mM (5 mg/ml) and stored at −80°C until use [37]. Before imaging, CTZ stock was diluted in Duelbecco's-PBS (D-PBS) to 1.2 mg/ml work solution. Mice were anesthetized by inhalation of 4% isofluorane and each administrated with 100 µl CTZ work solution by either *i.v.*, *i.p.,* or intra-tissue cage (*i.t*.) route, at a dose of 4 mg/kg body weight. *In vivo* imaging was performed at the indicated time after CTZ administration using an IVIS Spectrum system (Caliper Life Sciences, Alameda, CA). Mice were positioned within the imaging chamber and 2.5% isofluorane in air was administered *via* nose cones through the IXG8 gas anesthesia system (Caliper Life Sciences). Images used for quantification analysis for *i.t*. route administration of CTZ were all acquired using 16 second integration times. For assessment of *Gaussia* luminescence intensity at a given location by the Living image software (version 4.3.1), pseudocolor images were generated and represented with different colors according to the color scale bar (from blue, least intense, to red, most intense; units as photons second^−1^ cm^−2^ steradian^−1^). Total luminescence quantification (photons second^−1^) at each time point was done by the region of interest (ROI) tool of the Living image software.

### Measurement of Gluc activity in TCF and plasma

Clear supernatant of TCF or plasma samples (see the Experimental Procedure for sampling of TCF and plasma) was initially diluted 50-fold in D-PBS then further 1∶ 10 serially diluted. For each dilution, 50 µl sample was loaded into each well of 96-well plates (serial number 3912, Corning, NY). The plates were assessed by adding 50 µl of CTZ substrate to each well at the final concentration of either 118 µM or 39 µM *via* a built-in substrate injector in luminometer (Model of Infinite® 200 PRO, Tecan Systems, San Jose, CA). Gluc activity in TCF (CPS per µl TCF) or Gluc activity in plasma (CPS per µl plasma) was determined by the luminescence signal falling into the linear measurement range of the luminometer. It was found that 118 µM CTZ yield better results than 39 µM. Consequently, 118 µM CTZ was used for all final quantification analysis.

### Statistic Analysis

Unpaired and two tailed Student's *t*-test was used for all two comparisons with one variable (indicated by the connected lines in the figure), *P*<0.01 was considered as statistically significant.

## Supporting Information

Figure S1 **Gluc without secretion signal expressed by** ***E. coli*** **is associated with bacterial cell pellet but not culture supernatant.**
(TIF)

Figure S2 **Integration of the** ***pelB*** **tagged** ***gluc*** **gene in the same orientation as the original** ***lacZ*** **ORF on** ***E. coli*** **chromosome reproducibly generates higher level of secretion of Gluc to bacterial culture supernatant than other integration orientations.**
(TIF)

Figure S3 **Integration of the** ***pelB*** **tagged** ***gluc*** **gene into chromosomal** ***lacZ*** **locus is stable** ***in vitro.***
(TIF)

Figure S4 **Comparison of growth rate and bioluminescence production for a PelB tagged Gluc expressing strain ML6189 and the parental strain ATCC25922** ***in vitro.***
(TIF)

Figure S5 **Secreted Gluc activity in tissue cage fluid (TCF) correlates with CFU burden of the recombinant** ***E. coli*** **in TCF.**
(TIF)

File S1 **includes supplementary figure legend, primer sequences and plasmids used in this study.**
(DOCX)

## References

[1] Andreu N, Zelmer A, Wiles S (2011) Noninvasive biophotonic imaging for studies of infectious disease. FEMS Microbiol Rev 35: 360–394. 20955395 10.1111/j.1574-6976.2010.00252.x PMC3084502

[2] Andreu N, Zelmer A, Fletcher T, Elkington PT, Ward TH, et al (2010) Optimisation of bioluminescent reporters for use with mycobacteria. PLoS One 5: e10777. 20520722 10.1371/journal.pone.0010777 PMC2875389

[3] Andreu N, Zelmer A, Sampson SL, Ikeh M, Bancroft GJ, et al (2013) Rapid in vivo assessment of drug efficacy against Mycobacterium tuberculosis using an improved firefly luciferase. J Antimicrob Chemother 68: 2118–2127. 23633686 10.1093/jac/dkt155 PMC3743513

[4] Daniel C, Poiret S, Dennin V, Boutillier D, Pot B (2013) Bioluminescence imaging study of spatial and temporal persistence of Lactobacillus plantarum and Lactococcus lactis in living mice. Appl Environ Microbiol 79: 1086–1094. 23204409 10.1128/AEM.03221-12 PMC3568624

[5] Francis KP, Yu J, Bellinger-Kawahara C, Joh D, Hawkinson MJ, et al (2001) Visualizing pneumococcal infections in the lungs of live mice using bioluminescent Streptococcus pneumoniae transformed with a novel gram-positive lux transposon. Infect Immun 69: 3350–3358. 11292758 10.1128/IAI.69.5.3350-3358.2001 PMC98294

[6] Wurdinger T, Badr C, Pike L, de Kleine R, Weissleder R, et al (2008) A secreted luciferase for ex vivo monitoring of in vivo processes. Nat Methods 5: 171–173. 18204457 10.1038/nmeth.1177 PMC2699561

[7] Chung E, Yamashita H, Au P, Tannous BA, Fukumura D, et al (2009) Secreted Gaussia luciferase as a biomarker for monitoring tumor progression and treatment response of systemic metastases. PLoS One 4: e8316. 20016816 10.1371/journal.pone.0008316 PMC2789383

[8] Niers JM, Kerami M, Pike L, Lewandrowski G, Tannous BA (2011) Multimodal in vivo imaging and blood monitoring of intrinsic and extrinsic apoptosis. Mol Ther 19: 1090–1096. 21343914 10.1038/mt.2011.17 PMC3129810

[9] Santos EB, Yeh R, Lee J, Nikhamin Y, Punzalan B, et al (2009) Sensitive in vivo imaging of T cells using a membrane-bound Gaussia princeps luciferase. Nat Med 15: 338–344. 19219023 10.1038/nm.1930 PMC2837150

[10] Enjalbert B, Rachini A, Vediyappan G, Pietrella D, Spaccapelo R, et al (2009) A multifunctional, synthetic Gaussia princeps luciferase reporter for live imaging of Candida albicans infections. Infect Immun 77: 4847–4858. 19687206 10.1128/IAI.00223-09 PMC2772526

[11] Shao N, Bock R (2008) A codon-optimized luciferase from Gaussia princeps facilitates the in vivo monitoring of gene expression in the model alga Chlamydomonas reinhardtii. Curr Genet 53: 381–388. 18408930 10.1007/s00294-008-0189-7 PMC2413079

[12] Tannous BA, Kim DE, Fernandez JL, Weissleder R, Breakefield XO (2005) Codon-optimized Gaussia luciferase cDNA for mammalian gene expression in culture and in vivo. Mol Ther 11: 435–443. 15727940 10.1016/j.ymthe.2004.10.016

[13] Wiles S, Ferguson K, Stefanidou M, Young DB, Robertson BD (2005) Alternative luciferase for monitoring bacterial cells under adverse conditions. Appl Environ Microbiol 71: 3427–3432. 16000745 10.1128/AEM.71.7.3427-3432.2005 PMC1169068

[14] Green AA, McElroy WD (1956) Crystalline firefly luciferase. Biochim Biophys Acta 20: 170–176. 13315363 10.1016/0006-3002(56)90275-x

[15] Lembert N, Idahl LA (1995) Regulatory effects of ATP and luciferin on firefly luciferase activity. Biochem J 305 (Pt 3): 929–933. 10.1042/bj3050929 PMC1136347 7848294

[16] de Wet JR, Wood KV, Helinski DR, DeLuca M (1985) Cloning of firefly luciferase cDNA and the expression of active luciferase in Escherichia coli. Proc Natl Acad Sci U S A 82: 7870–7873. 3906652 10.1073/pnas.82.23.7870 PMC390871

[17] Meighen EA (1993) Bacterial bioluminescence: organization, regulation, and application of the lux genes. FASEB J 7: 1016–1022. 8370470 10.1096/fasebj.7.11.8370470

[18] Koga K, Harada T, Shimizu H, Tanaka K (2005) Bacterial luciferase activity and the intracellular redox pool in Escherichia coli. Mol Genet Genomics 274: 180–188. 16047200 10.1007/s00438-005-0008-5

[19] Galluzzi L, Karp M (2007) Intracellular redox equilibrium and growth phase affect the performance of luciferase-based biosensors. J Biotechnol 127: 188–198. 16891024 10.1016/j.jbiotec.2006.06.019

[20] Wille T, Blank K, Schmidt C, Vogt V, Gerlach RG (2012) Gaussia princeps luciferase as a reporter for transcriptional activity, protein secretion, and protein-protein interactions in Salmonella enterica serovar typhimurium. Appl Environ Microbiol 78: 250–257. 22020521 10.1128/AEM.06670-11 PMC3255616

[21] Bamberger DM, Peterson LR, Gerding DN, Moody JA, Fasching CE (1986) Ciprofloxacin, azlocillin, ceftizoxime and amikacin alone and in combination against gram-negative bacilli in an infected chamber model. J Antimicrob Chemother 18: 51–63. 10.1093/jac/18.1.51 3093445

[22] Blaser J, Vergeres P, Widmer AF, Zimmerli W (1995) In vivo verification of in vitro model of antibiotic treatment of device-related infection. Antimicrob Agents Chemother 39: 1134–1139. 7625801 10.1128/aac.39.5.1134 PMC162696

[23] Zimmerli W, Lew PD, Waldvogel FA (1984) Pathogenesis of foreign body infection. Evidence for a local granulocyte defect. J Clin Invest 73: 1191–1200. 6323536 10.1172/JCI111305 PMC425133

[24] Lobo LA, Jenkins AL, Jeffrey Smith C, Rocha ER (2013) Expression of Bacteroides fragilis hemolysins in vivo and role of HlyBA in an intra-abdominal infection model. Microbiologyopen 2: 326–337. 23441096 10.1002/mbo3.76 PMC3633356

[25] Kristian SA, Golda T, Ferracin F, Cramton SE, Neumeister B, et al (2004) The ability of biofilm formation does not influence virulence of Staphylococcus aureus and host response in a mouse tissue cage infection model. Microb Pathog 36: 237–245. 15043859 10.1016/j.micpath.2003.12.004

[26] Kristian SA, Lauth X, Nizet V, Goetz F, Neumeister B, et al (2003) Alanylation of teichoic acids protects Staphylococcus aureus against Toll-like receptor 2-dependent host defense in a mouse tissue cage infection model. J Infect Dis 188: 414–423. 12870123 10.1086/376533

[27] Dawson J, Rordorf-Adam C, Geiger T, Towbin H, Kunz S, et al (1993) Interleukin-1 (IL-1) production in a mouse tissue chamber model of inflammation. II. Identification of (tissue) macrophages as the IL-1 producing cells and the effect of anti-inflammatory drugs. Agents Actions 38: 255–264. 8213352 10.1007/BF01976218

[28] Bamberger DM, Herndon BL, Fitch J, Florkowski A, Parkhurst V (2002) Effects of neutrophils on cefazolin activity and penicillin-binding proteins in Staphylococcus aureus abscesses. Antimicrob Agents Chemother 46: 2878–2884. 12183241 10.1128/AAC.46.9.2878-2884.2002 PMC127421

[29] McCallum N, Karauzum H, Getzmann R, Bischoff M, Majcherczyk P, et al (2006) In vivo survival of teicoplanin-resistant Staphylococcus aureus and fitness cost of teicoplanin resistance. Antimicrob Agents Chemother 50: 2352–2360. 16801412 10.1128/AAC.00073-06 PMC1489778

[30] Lucet JC, Herrmann M, Rohner P, Auckenthaler R, Waldvogel FA, et al (1990) Treatment of experimental foreign body infection caused by methicillin-resistant Staphylococcus aureus. Antimicrob Agents Chemother 34: 2312–2317. 2128441 10.1128/aac.34.12.2312 PMC172053

[31] Li C, Nicolau DP, Lister PD, Quintiliani R, Nightingale CH (2004) Pharmacodynamic study of beta-lactams alone and in combination with beta-lactamase inhibitors against Pseudomonas aeruginosa possessing an inducible beta-lactamase. J Antimicrob Chemother 53: 297–304. 14729755 10.1093/jac/dkh057

[32] Fernandez J, Barrett JF, Licata L, Amaratunga D, Frosco M (1999) Comparison of efficacies of oral levofloxacin and oral ciprofloxacin in a rabbit model of a staphylococcal abscess. Antimicrob Agents Chemother 43: 667–671. 10049285 10.1128/aac.43.3.667 PMC89178

[33] Bamberger DM, Herndon BL, Suvarna PR (1995) Azithromycin in an experimental Staphylococcus aureus abscess model. J Antimicrob Chemother 35: 623–629. 7592175 10.1093/jac/35.5.623

[34] Anderson JC, Dueber JE, Leguia M, Wu GC, Goler JA, et al (2010) BglBricks: A flexible standard for biological part assembly. J Biol Eng 4: 1. 20205762 10.1186/1754-1611-4-1 PMC2822740

[35] Datsenko KA, Wanner BL (2000) One-step inactivation of chromosomal genes in Escherichia coli K-12 using PCR products. Proc Natl Acad Sci U S A 97: 6640–6645. 10829079 10.1073/pnas.120163297 PMC18686

[36] Maguire CA, Bovenberg MS, Crommentuijn MH, Niers JM, Kerami M, et al (2013) Triple bioluminescence imaging for in vivo monitoring of cellular processes. Mol Ther Nucleic Acids 2: e99. 23778500 10.1038/mtna.2013.25 PMC3696905

[37] Tannous BA (2009) Gaussia luciferase reporter assay for monitoring biological processes in culture and in vivo. Nat Protoc 4: 582–591. 19373229 10.1038/nprot.2009.28 PMC2692611

[38] Inouye S, Sahara Y (2008) Identification of two catalytic domains in a luciferase secreted by the copepod Gaussia princeps. Biochem Biophys Res Commun 365: 96–101. 17981153 10.1016/j.bbrc.2007.10.152

[39] Maguire CA, Deliolanis NC, Pike L, Niers JM, Tjon-Kon-Fat LA, et al (2009) Gaussia luciferase variant for high-throughput functional screening applications. Anal Chem 81: 7102–7106. 19601604 10.1021/ac901234r PMC2846205

[40] Contag CH (2007) In vivo pathology: seeing with molecular specificity and cellular resolution in the living body. Annu Rev Pathol 2: 277–305. 18039101 10.1146/annurev.pathol.2.010506.091930

[41] Morse D, Tannous BA (2012) A water-soluble coelenterazine for sensitive in vivo imaging of coelenterate luciferases. Mol Ther 20: 692–693. 22472977 10.1038/mt.2012.38 PMC3321601

[42] Degeling MH, Bovenberg MS, Lewandrowski GK, de Gooijer MC, Vleggeert-Lankamp CL, et al (2013) Directed molecular evolution reveals Gaussia luciferase variants with enhanced light output stability. Anal Chem 85: 3006–3012. 23425213 10.1021/ac4003134 PMC3617556

[43] Welsh JP, Patel KG, Manthiram K, Swartz JR (2009) Multiply mutated Gaussia luciferases provide prolonged and intense bioluminescence. Biochem Biophys Res Commun 389: 563–568. 19825431 10.1016/j.bbrc.2009.09.006

[44] Kim SB, Suzuki H, Sato M, Tao H (2011) Superluminescent variants of marine luciferases for bioassays. Anal Chem 83: 8732–8740. 21951281 10.1021/ac2021882

[45] Boyle VJ, Fancher ME, Ross RW Jr (1973) Rapid, modified Kirby-Bauer susceptibility test with single, high-concentration antimicrobial disks. Antimicrob Agents Chemother 3: 418–424. 4790600 10.1128/aac.3.3.418 PMC444425

[46] Wessel D, Flugge UI (1984) A method for the quantitative recovery of protein in dilute solution in the presence of detergents and lipids. Anal Biochem 138: 141–143. 6731838 10.1016/0003-2697(84)90782-6

[47] Morris CE, Klement JF, McAllister WT (1986) Cloning and expression of the bacteriophage T3 RNA polymerase gene. Gene 41: 193–200. 3011596 10.1016/0378-1119(86)90098-3

[48] Dawson J, Rordorf-Adam C, Geiger T, Towbin H, Kunz S, et al (1993) Interleukin-1 (IL-1) production in a mouse tissue chamber model of inflammation. I. Development and initial characterisation of the model. Agents Actions 38: 247–254. 8213351 10.1007/BF01976217

[49] Golde WT, Gollobin P, Rodriguez LL (2005) A rapid, simple, and humane method for submandibular bleeding of mice using a lancet. Lab Anim (NY) 34: 39–43. 10.1038/laban1005-39 16195737

