# Supplementary figures and images for: Secreted Gaussia princeps Luciferase as a Reporter of Escherichia coli Replication in a Mouse Tissue Cage Model of Infection
Source: PLoS One. 2014 Mar 4;9(3):e90382. doi: 10.1371/journal.pone.0090382 (PMC3942414; doi:10.1371/journal.pone.0090382)

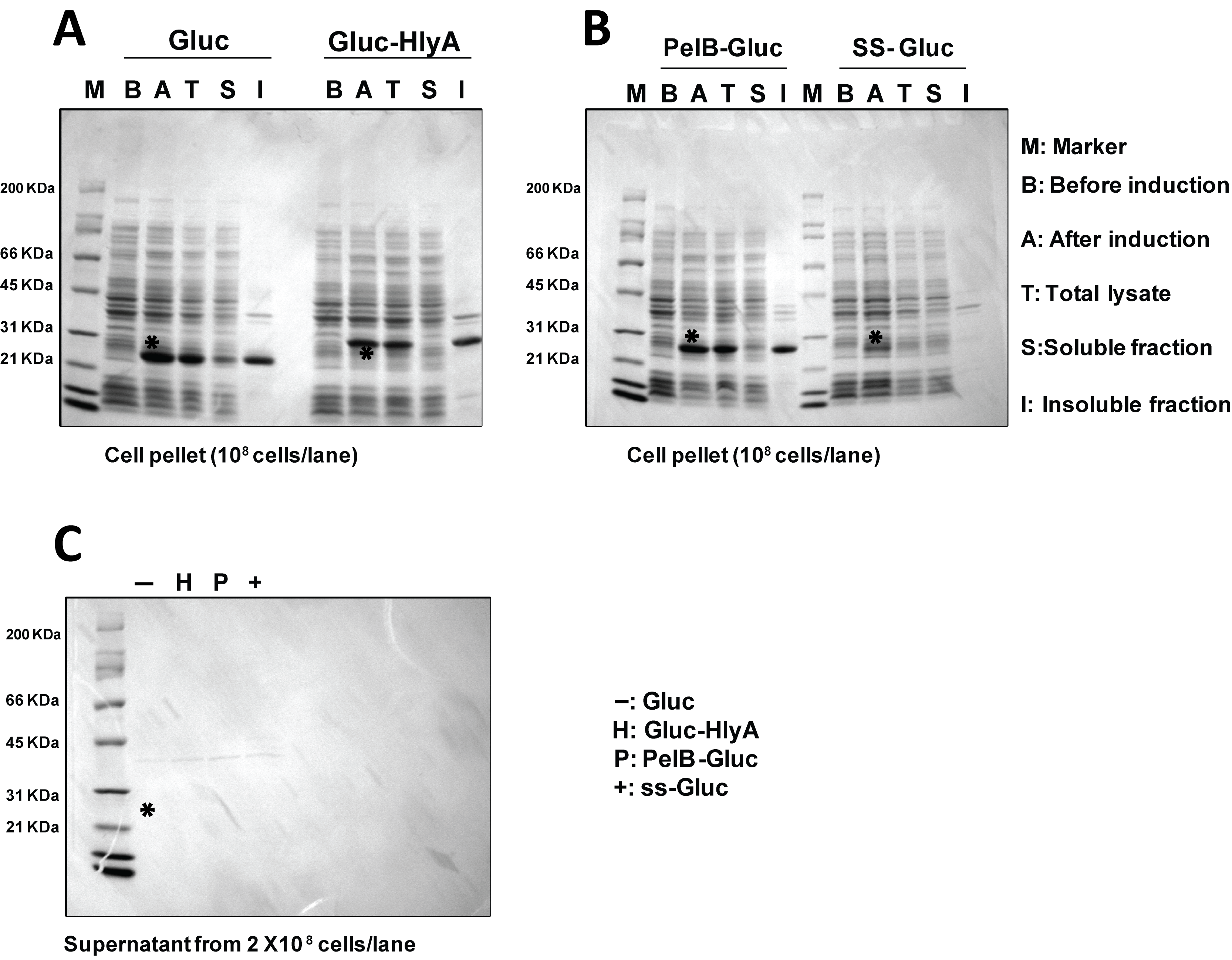

Supplement: Figure S1 — Gluc without secretion signal expressed by E. coli is associated with bacterial cell pellet but not culture supernatant. (TIF) [file pone.0090382.s001.tif]

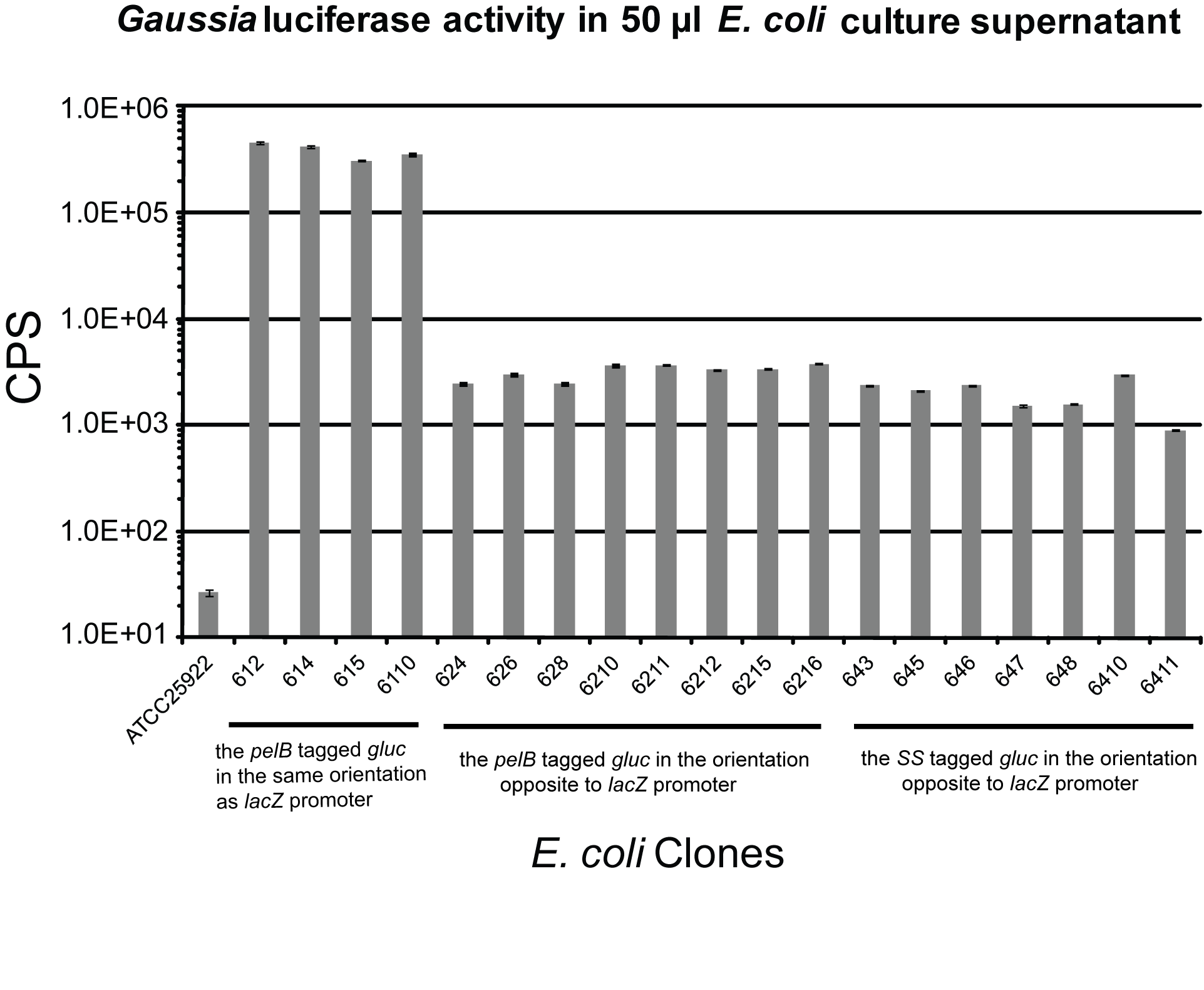

Supplement: Figure S2 — Integration of the pelB tagged gluc gene in the same orientation as the original lacZ ORF on E. coli chromosome reproducibly generates higher level of secretion of Gluc to bacterial culture supernatant than other integration orientations. (TIF) [file pone.0090382.s002.tif]

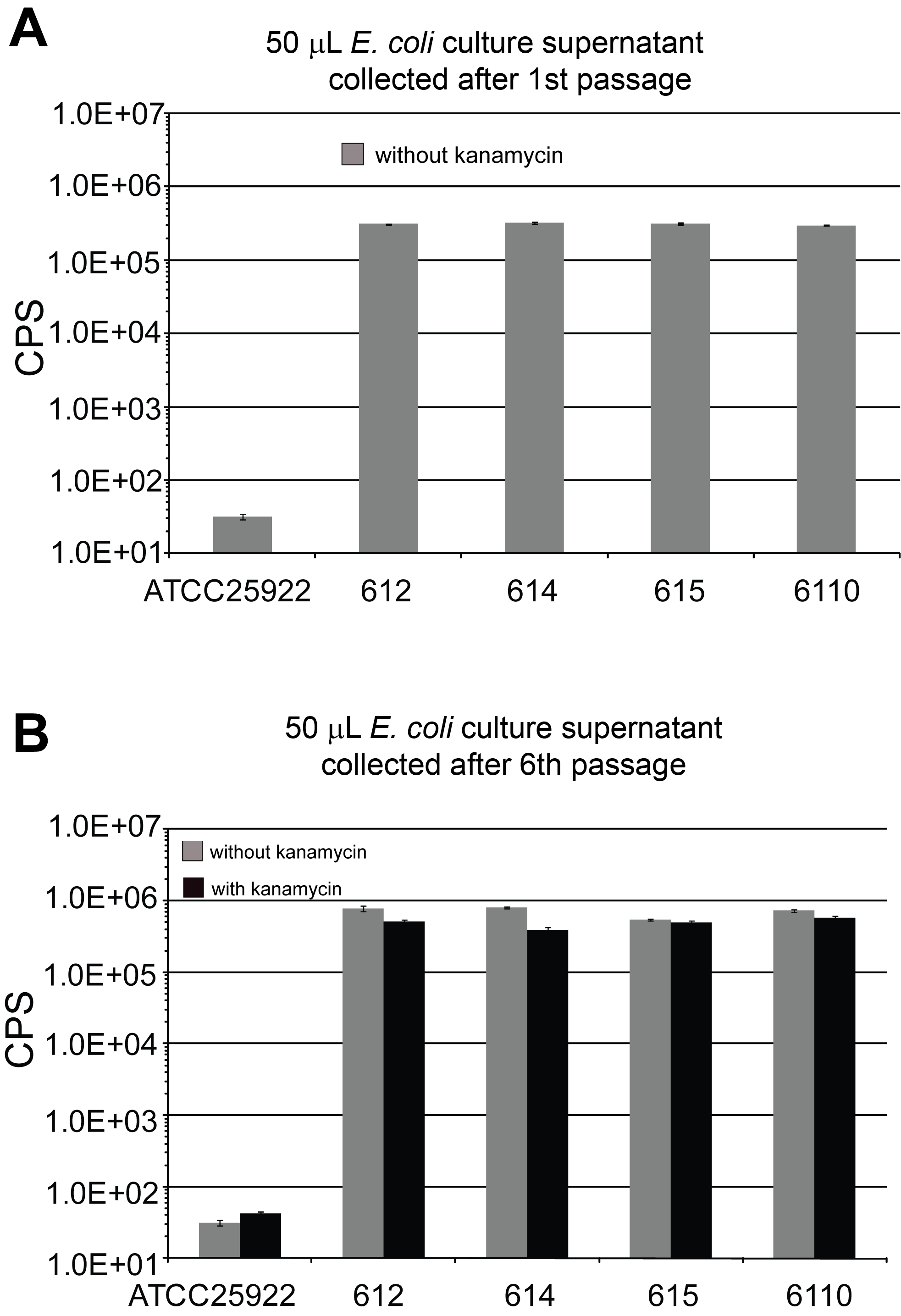

Supplement: Figure S3 — Integration of the pelB tagged gluc gene into chromosomal lacZ locus is stable in vitro. (TIF) [file pone.0090382.s003.tif]

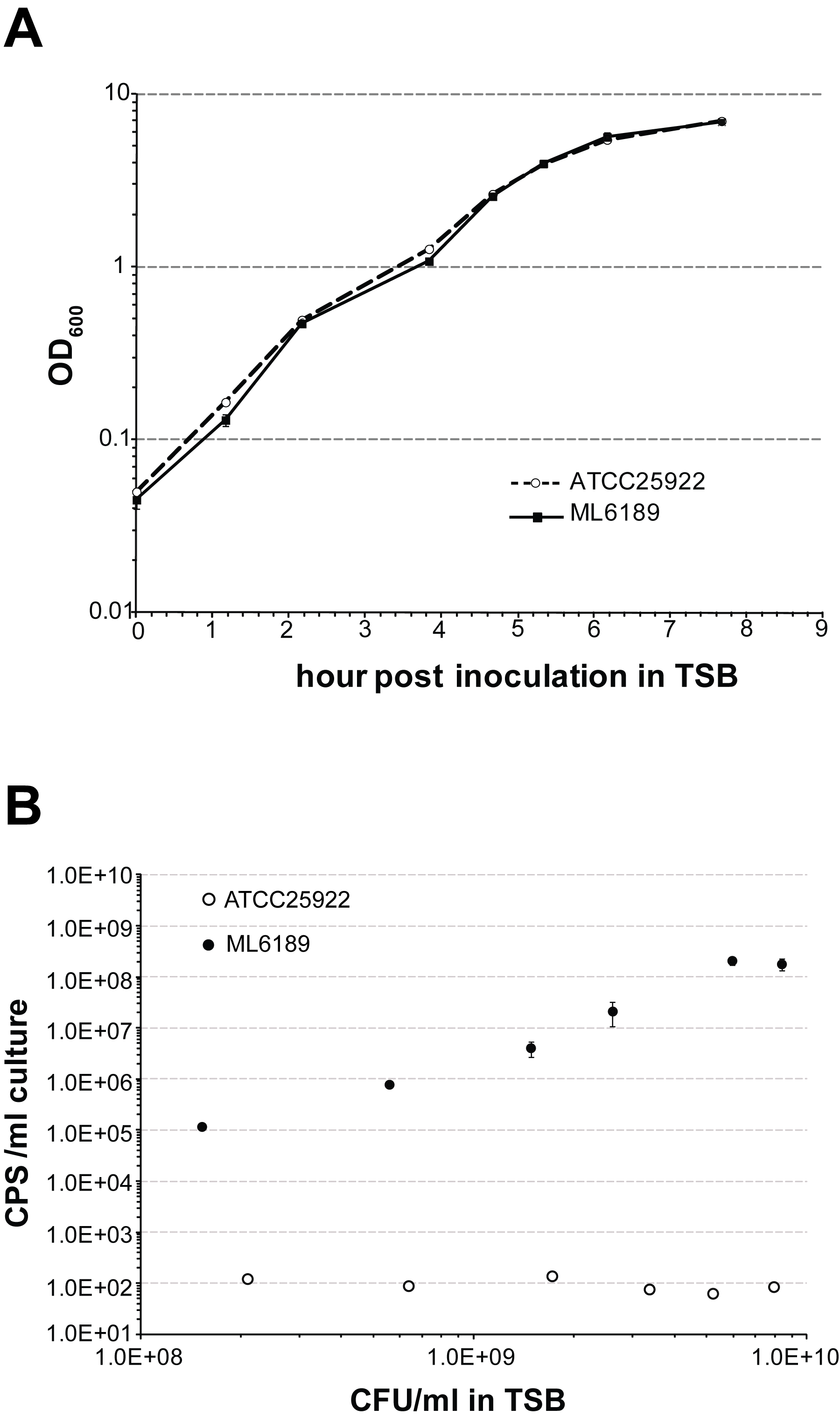

Supplement: Figure S4 — Comparison of growth rate and bioluminescence production for a PelB tagged Gluc expressing strain ML6189 and the parental strain ATCC25922 in vitro. (TIF) [file pone.0090382.s004.tif]

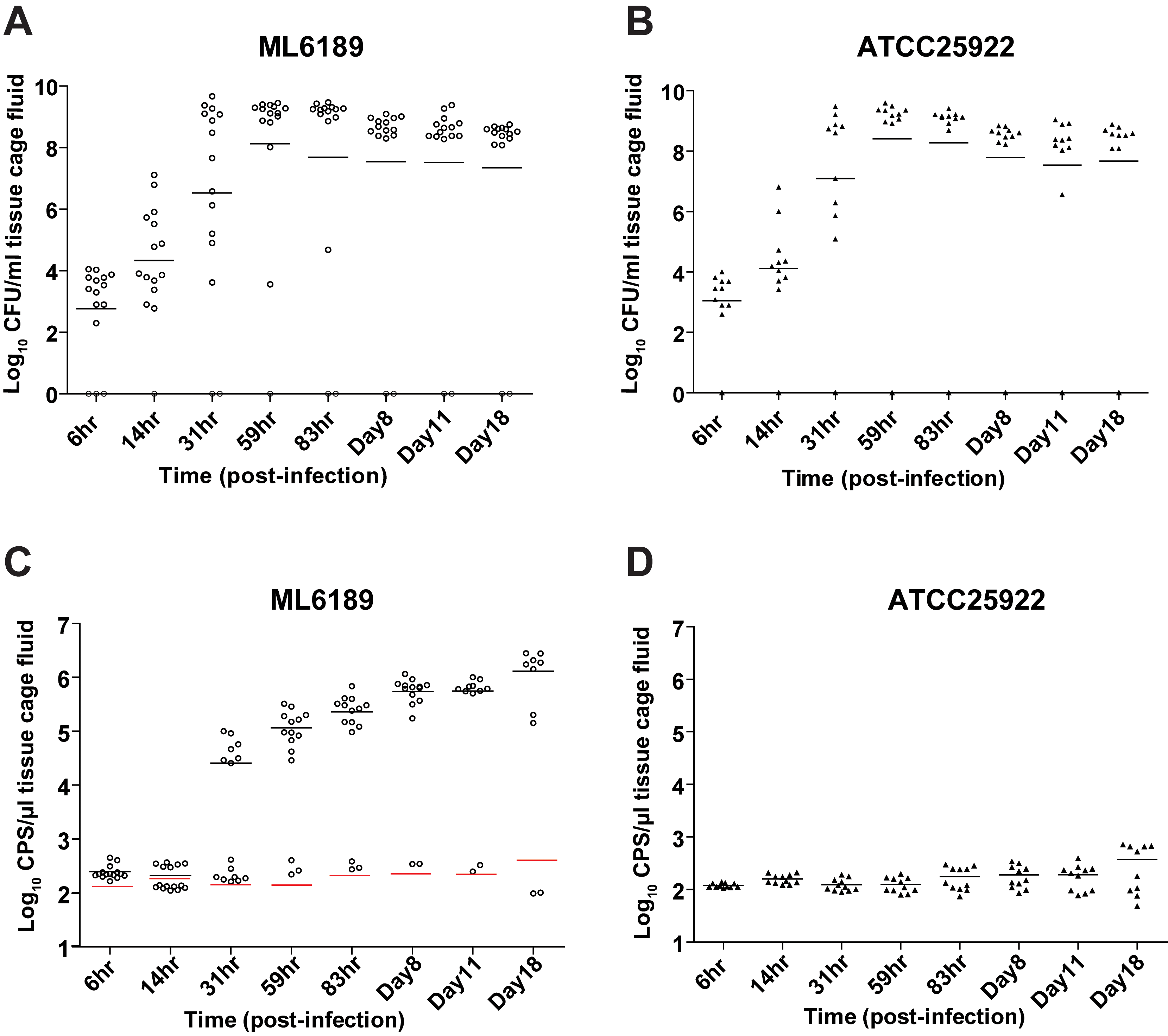

Supplement: Figure S5 — Secreted Gluc activity in tissue cage fluid (TCF) correlates with CFU burden of the recombinant E. coli in TCF. (TIF) [file pone.0090382.s005.tif]
