# Supplementary material for: Secreted Gaussia princeps Luciferase as a Reporter of Escherichia coli Replication in a Mouse Tissue Cage Model of Infection
Source: PLoS One. 2014 Mar 4;9(3):e90382. doi: 10.1371/journal.pone.0090382 (PMC3942414; doi:10.1371/journal.pone.0090382)
Supplement: File S1 — includes supplementary figure legend, primer sequences and plasmids used in this study. (DOCX) [file pone.0090382.s006.docx]

**Supplementary figure legend**

**Fig. S1 Gluc without secretion signal expressed by *E. coli* is associated with bacterial cell pellet but not culture supernatant.**

M: lane for protein marker, B: whole *E. coli* cell lysate before induction with IPTG, A: whole cell lysate after induction, T: total whole cell lysate after French press lysis, S: soluble fraction of lysate after French press, I: insoluble fraction of lysate after French press. *: the predicted position of Gluc or Gluc fusion protein on protein gel. For panel A and B, lysate corresponding to 10^8^ cells was loaded in each lane. (A) The expression of Gluc without secretion signal (Gluc) or Gluc with C-terminal secretion signal derived from α-hemolysin (Gluc-HlyA) was induced from pCOLDI-based vectors in *E. coli.* Expressions were detected by Coomassie Blue staining. (B) The expression of Gluc with N-terminal secretion signal derived from pectate lyase (PelB-Gluc) or Gluc with its native secretion signal from *Gaussia princeps* (SS-Gluc) was induced from pCOLDI-based vectors in *E. coli* and detected by Coomassie Blue staining. (C) ­For panel C, the bacterial culture supernatant corresponding to secreted protein from 2 X 10^8^ cells was loaded in each lane. −: Culture supernatant of *E. coli* expressing Gluc without secretion signal, H: supernatant of *E. coli* expressing Gluc-HlyA, P: supernatant of *E. coli* expressing PelB-Gluc, +: supernatant of *E. coli* expressing SS-Gluc.

**Fig. S2 Integration of the *pelB* tagged *gluc* gene in the same orientation as the original *lacZ* ORF on *E. coli* chromosome reproducibly generates higher level of secretion of Gluc to bacterial culture supernatant than other integration orientations*.***

All *E. coli* clones were grown in LB medium without kanamycin supplement overnight and 50 µl culture supernatant was measured for secreted Gluc activity. Note: all results have been normalized with OD_600_. CPS: photon counts per second. *E. coli* strain ATCC25922 is the negative control strain without integration of *gluc* gene on the chromosome. The category of the clones, indicating identity of the secretion tag and its orientation relative to the *LacZ* promoter are below the clone number. Data represent mean and standard error of triplicate samples. Error bars may be too small to see for some samples.

**Fig. S3 Integration of the *pelB* tagged *gluc* gene into chromosomal *lacZ* locus is stable *in vitro.***

All results have been normalized against OD_600_ of the culture. For both panels, data represent mean and standard error of triplicate samples. Error bars may be too small to see for some samples. CPS: photon counts per second. Clones other than the parental strain ATCC25922, harbor the *pelB* tagged *gluc* gene integrated in the same orientation as *LacZ*. All clones harbor the kanamycin resistance gene accompanying the *pelB* tagged *gluc* gene. (A) *E. coli* clones with the *pelB* tagged *gluc* gene were grown in LB medium without kanamycin for one passage and measured for the secretion of Gluc into culture supernatant. (B) *E. coli* clones from panel A have undergone 5 more serial passages either in media supplied with kanamycin (black bar) or without kanamycin (grey bar). Culture supernatant corresponding to the 6^th^ passage was measured for the secretion of Gluc.

**Fig. S4 Comparison of growth rate and bioluminescence production for a PelB tagged Gluc expressing strain ML6189 and the parental strain ATCC25922 *in vitro.***

For both panels, data represent mean and standard error of triplicate samples. Error bars may be too small to see for some samples. CPS: photon counts per second. (A) The recombinant *E .coli* strain ML6189 expressing the PelB tagged Gluc (solid line) and the parental strain ATCC25922 (dashed line) show similar growth curves in Tryptic Soy Broth (TSB) medium. (B) Gluc bioluminescence (CPS) and colony forming units (CFU) were measured from 2 hour post inoculation (exponential phase) to 8 hour post inoculation (stationary phase) for the cultures in panel A*.*

**Fig. S5 Secreted Gluc activity in tissue cage fluid (TCF) correlates with CFU burden of the recombinant *E. coli* in TCF.**

Mouse tissue cages were inoculated with 10^3^ CFU of the recombinant *E. coli* strain ML6189 expressing Gluc or the parental strain ATCC25922. TCF was collected at different time points of infection and analyzed by both CFU plating and measurement of secreted Gluc activity (see Experimental Procedures). CPS: photon counts per second. For all panels, the mean value for each group of samples is represented by a black bar. Data from two independent experiments were pooled. (A) CFU burden of ML6189 in TCF at different time points during 18 days of tissue cage infection. Note: data points plotted on the X-axis indicate mice without detectable CFU at the corresponding time points. (B) CFU burden of ATCC25922 in TCF at different time points along 18 days of tissue cage infection. Note: no detectable CFU was recovered from a single animal at all time points. (C) Secreted Gluc activity in TCF at different time points for ML6189 infection. The red bars represent mean value of the background Gluc signal calculated from data in panel D. Note: for ML6189 infection, 4 mice (day 11) and 5 mice (day 18) were removed from the group for imaging experiments and were excluded from quantification of Gluc activity in TCF (panel C). (D) Secreted Gluc activity in TCF at different time points for ATCC25922 infection.

**List S1 Primers and promoter sequences used in this paper (sequence 5’🡪3’)**

**Primers for making pSMM25 plasmid**

Smm105 gcagcattacacgtctggatccattgtgtaggctggagc

Smm106 gctccagcctacacaatggatccagacgtgtaatgctgc

Smm107 gcttgcatgcagattgcagaattccacgtctggatccattg

Smm108 caatggatccagacgtggaattctgcaatctgcatgcaagc

**Primers for PCR DNA fragments from template plasmids**

FlacZ_ForwardL_MYL ATGACCATGA TTACGGATTC ACTGGCCGTC GTTTTACAAC G GCATGCATG C AGATTGCAGA

FlacZ_ReverseL_MYL TTATTTTTGA CACCAGACCA ACTGGTAATG GTAGCGACCG G CATATGAATA TCCTCCTTAG

OlacZ_ForwardL_MYL TTATTTTTGA CACCAGACCA ACTGGTAATG GTAGCGACCG G GCATGCATG C AGATTGCAGA

OlacZ_ReverseL_MYL ATGACCATGA TTACGGATTC ACTGGCCGTC GTTTTACAAC G CATATGAATA TCCTCCTTAG

**Primers for PCR validation of template plasmids and *gluc* integration on the chromosome**

MYL-KanR-k1 CAGTCATAGCCGAATAGCCT

MYL-KanR-k2 CGGTGCCCTGAATGAACTGC

MYL-KanR-kt CGGCCACAGTCGATGAATCC

MYL-PelB-P1 ATGAA ATACCTGCTG CCGAC

MYL-SS-P1 GG TGTTAAAGTT CTGTTCGC

MYL-pKD4-P2 CATATGAATATCCTCCTTAG

JW-Gluc GT R1 AACTGTTCCA TCGGTTCCAG

MYL-lacZF1 ACG TCG TGA CTG GGA AAA CC

MYL-BHR5-H1 TGTTCCCACGGAGAATCCGA

MYL-AmpR-Forward AACTCTCAAGGATCTTACCG

MYL-AmpR-reverse CAGGCAACTATGGATGAACG

**Promoter sequence with proposed strong strength**

TTGACAGCTA GCTCAGTCCT AGGTACTGTG CTAGCTACTA GAGAAAGAGG AGAAATACCA TATG

**promoter sequence with proposed intermediate strength**

TTTATGGCTA GCTCAGTCCT AGGTACAATG CTAGCTACTA GAGAAAGAGG AGAAATACCA TATG

**promoter sequence with proposed moderate strength**

CTGATGGCTA GCTCAGTCCT AGGGATTATG CTAGCTACTA GAGAAAGAGG AGAAATACCA TATG

**Sequencing primers for confirmation of template plasmids**

MYL_Seq_3878 TGGCCAGTGCCAAGCTTGCATGC

MYL-Seq_479 AACAGTTCATCGCGCAGGTTGAC

MYL_seq_198R GTTAGACGCAACCGCAACGATGTT

MYL_seq_272R TCTTTCAGAACTTCCAGCGGCAG

MYL_seq_100R TCGGCAGCAGGTATTTCATA

**List S2 Plasmids used in this study**

**Plasmid Characteristic(s) Source or Reference**

pKD46 λ Red-recombinase under control of Pc *P_araB_,* [[1](#_ENREF_1)]

R101 origin, temperature-sensitive, Amp-resistant

pKD4 contain an FRT-flanked kanamycin resistance gene [[1](#_ENREF_1)]

R6Kγ origin, Amp-resistant

pSMM25 pKD4 containing BamH I and Eco RI sites this study

GS50065-1 strong strength promoter cloned into pBlueScript (II) SK (-) vector this study

GS50065-2 intermediate strength promoter cloned into pBlueScript (II) SK (-) vector this study

GS50065-3 moderate strength promoter cloned into pBlueScript (II) SK (-) vector this study

GS4624-1 synthetic *E. coli* codon-optimized *gluc* gene without secretion signal this study

cloned into pBlueScript (II) SK (-) vector

GS4624-2 synthetic *E. coli* codon-optimized *gluc* gene with C-terminal this study

HlyA secretion signal cloned into pBlueScript (II) SK (-) vector

GS4624-4 synthetic *E. coli* codon-optimized *gluc* gene with N-terminal this study

PelB secretion signal cloned into pBlueScript (II) SK (-) vector

GS50101 synthetic *E. coli* codon-optimized *gluc* gene with N-terminal this study

native secretion signal (SS) cloned into pBlueScript (II) SK (-) vector

pCOLDI *cspA* promoter, ColE1 origin, Amp-resistant , *lacI*  [[2](#_ENREF_2)]

pCOLDI (Gluc) His-tagged synthetic *E. coli* codon-optimized *gluc* gene without secretion signal this study

cloned into pCOLDI expression vector

pCOLDI (Gluc-HlyA) His-tagged synthetic *E. coli* codon-optimized *gluc* gene with C-terminal this study

HlyA secretion signal cloned into pCOLDI expression vector

pCOLDI (PelB-Gluc) His-tagged synthetic *E. coli* codon-optimized *gluc* gene with N-terminal this study

PelB secretion signal cloned into pCOLDI expression vector

pCOLDI (SS-Gluc) His- tagged synthetic *E. coli* codon-optimized *gluc* gene with N-terminal this study

native secretion signal (SS) cloned in pCOLDI expression vector

pJWW3 (moderate promoter driven SS-Gluc, template plasmid)

moderate strength promoter driven synthetic *E. coli* codon-optimized *gluc* gene with N-terminal this study

native secretion signal cloned into pSMM25 vector

pJWW6 (moderate promoter driven PelB-Gluc, template plasmid)

moderate strength promoter driven synthetic *E. coli* codon-optimized *gluc* gene with N-terminal this study

PelB secretion signal cloned into pSMM25 vector

pCP20 Amp-resistant, chloramphenicol -resistant [[3](#_ENREF_3)]

temperature-sensitive and Flippase expression

**List S3 *E. coli* Strains/Clones generated by this study**

**Strain Relevant characteristic(s) Source/Reference**

612 *pelB* tagged *gluc* gene replacing chromosomal *lacZ* ORF

with the same orientation as original *LacZ*, *Kan^R^*  this study

614 same as the above this study

615 same as the above this study

6110 same as the above this study

624 *pelB* tagged *gluc* gene replacing chromosomal *lacZ* ORF

with the orientation opposite to original *LacZ, Kan^R^* this study

626 same as the above this study

628 same as the above this study

6210 same as the above this study

6211 same as the above this study

6212 same as the above this study

6215 same as the above this study

6216 same as the above this study

643 native secretion signal (SS) tagged *gluc* gene replacing chromosomal *lacZ* ORF

with the orientation opposite to original *LacZ, Kan^R^* this study

645 same as the above this study

646 same as the above this study

647 same as the above this study

648 same as the above this study

6410 same as the above this study

6411 same as the above this study

R7 *pelB* tagged *gluc* gene inserting into chromosomal *lacZ* ORF

with the same orientation as *lacZ, Kan^R^* this study

R76 *pelB* tagged *gluc* gene inserting into chromosomal *lacZ* ORF

with the same orientation as *lacZ, Kan^S^* this study

R79 same as the above this study

**Reference**

1. Datsenko KA, Wanner BL (2000) One-step inactivation of chromosomal genes in Escherichia coli K-12 using PCR products. Proc Natl Acad Sci U S A 97: 6640-6645.

2. Rathnayaka T, Tawa M, Sohya S, Yohda M, Kuroda Y (2010) Biophysical characterization of highly active recombinant Gaussia luciferase expressed in Escherichia coli. Biochim Biophys Acta 1804: 1902-1907.

3. Cherepanov PP, Wackernagel W (1995) Gene disruption in Escherichia coli: TcR and KmR cassettes with the option of Flp-catalyzed excision of the antibiotic-resistance determinant. Gene 158: 9-14.
